# Supplementary material for: Using telemedicine to improve access, cost and quality of secondary care for people in prison in England: a hybrid type 2 implementation effectiveness study
Source: BMJ Open. 2020 Feb 18;10(2):e035837. doi: 10.1136/bmjopen-2019-035837 (PMC7044812; doi:10.1136/bmjopen-2019-035837)
Supplement: Supplementary data [file bmjopen-2019-035837supp001.pdf]

STAFF TOPIC GUIDE PRISON TELEMEDICINE – Prior to telemedicine implementation

Introduction, consent taken

Organisational/ inter-relationships

- How does prison health/secondary care access fit into your organisational priorities/culture/norms?
- How does telemedicine fit into your organisational priorities/culture/norms?
- Thinking about national or local policies now, how do they influence your decision to introduce telemedicine?
- How networked would you say your organisation is with (relevant prisons/hospital)?

General background to telemedicine in prisons

We are planning to introduce telemedicine in HMP xxxx.

- What are your views on/what do you think about telemedicine?
- Why do you think we are planning on introducing telemedicine?
    - *FOR HOSPITALS ONLY – do you know of any barriers to accessing secondary care for prisoners which might influence the decision to introduce telemedicine ?*

Telemedicine in practice – working perspective

- How do you perceive your role in progressing telemedicine between hospitals and prisons?
    - *How does this fit with your current role?*
- Talk me through how you think a telemedicine consultation might work in practice
    - *What are the pros and cons of this consultation?*
    - *What else has to happen to make sure this consultation takes place successfully?*
- How might telemedicine impact on the work of other people in your organisation?
- How might your organisation react to this type of innovation? – why?
    - *What about the wider political/ professional system?*
- What has been your experience of introducing telemedicine so far?
- If we were to launch telemedicine tomorrow, is there anything else you think would get in the way?

Version 1 Date: 20/12/2019
Staff interview topic guide – prior to telemedicine implementation

Telemedicine benefits

- How might telemedicine affect you and your role?
- How might telemedicine affect patients in prisons?
- How might telemedicine affect your wider organisation (as opposed to just you or the patient)?
- How might telemedicine affect other associated partners (e.g. HMPPS/NHSE)?
- How might telemedicine affect you personally? (eg Extrinsic incentives such as goal-sharing awards, performance reviews, promotions, and raises in salary, and less tangible incentives such as increased stature or respect).

Telemedicine in practice – technological perspective

- What would an ideal system look like technology-wise?
- How easy or hard is it to work with other departments in your organisation to get the technology set up and ready to use?
- What might make people reluctant to use the telemedicine system (technology wise)?

Wrap up

Is there anything else you would like to add about prison-hospital telemedicine?

Thank and close

Version 1 Date: 20/12/2019
Staff interview topic guide – prior to telemedicine implementation
