# Supplementary material for: Using telemedicine to improve access, cost and quality of secondary care for people in prison in England: a hybrid type 2 implementation effectiveness study
Source: BMJ Open. 2020 Feb 18;10(2):e035837. doi: 10.1136/bmjopen-2019-035837 (PMC7044812; doi:10.1136/bmjopen-2019-035837)
Supplement: Supplementary data [file bmjopen-2019-035837supp002.pdf]

Using Video Consultations for Hospital Appointments in Prison – Focus Group Topic Guide

Introduction:

We are running a research project to see if using video consultations for hospital appointments in prisons makes healthcare better.

It can take a long time to get seen by a doctor at the hospital if you are in prison because only a few patients can go off-site every day. If we can do some appointments over the internet using a webcam patients might get seen quicker. It would also leave the hospital transfers for people who can't be seen over video consultation. We want to test whether prisons can do hospital appointments using video consultation. We want to know if this makes healthcare better for patients, and whether it helps the NHS too.

There are no plans we know of to try and use video consultations in HMP xxx at the moment, but we would like to gather your thoughts and feedback on this idea. We want to know what you would be worried about and how you think it should be used to make it a good experience.

IRAS study number: 229646, Version 1.1, 03/10/19

Timing	Section	Notes
	**Focus Group Opener**	
10 mins	I'd like to start with a bit of discussion around video consultations in prison.<br><br>**What were your initial thoughts when you first heard about the topic of this group?**<br><br>**How would you feel if you were offered a video consultation tomorrow?**<br><br>Essentially video appointments will be similar to the video court link process. You'll get a choice of whether to use the video system at the prison or wait for a face to face appointment.<br><br>**What is the best way we could name/describe these appointments?**	*after discussion provide further detail around what these consultations will hypothetically look like (e.g healthcare staff member in the room) and how appointments will be booked etc*
	**Concerns and benefits**	
30 mins	**What are your initial thoughts on video appointments for hospital care?**<br><br>• **What do you think people will see as benefits of video appointments?**<br><br>• **What do you think people will be concerned about in regards to video appointments?**<br><br>• **(if not already covered) Why do you think some people might refuse to have one?**	*After discussion if not already brought up, explain other health benefits e.g. shorter appointment waits, no prison officer in consultation, more dignity*
	**When would/wouldn't you choose it?**	
20 mins	We understand that even if people are generally OK with the idea of video appointments, there will be some circumstances where they still want to choose a face to face appointment.<br><br>**When might people choose to have a video appointment?**	*e.g. feeling really bad and want a quicker appointment, been waiting ages already, less*

IRAS study number: 229646, Version 1.1, 03/10/19

	**When might people choose not to have a video appointment?**	*worrisome health condition, private condition that you don't want PO to hear about*<br><br>*e.g. certain health conditions*
	**Opting in to telemedicine appointments**	
20 mins	People will always have the choice of having a normal face to face appointment if they get offered a video appointment. We want people to make a truly informed choice about which is best for them.<br><br>**Based on what we have just discussed, what do people need to know about video appointments to make an informed choice to have one or not?**<br><br>• **Do you think people will have confidence in the system?**<br><br>• **What would give people confidence in the system?**<br><br>**If people decide to opt for a video appointment, what information do they need before they have the actual appointment?**<br><br>**How could we best promote video appointments?**<br><br>**How should we provide 'mythbusting' information about them?**	*e.g. privacy, computer will work, Dr can diagnose ok over internet?*<br><br>*e.g. peer champions, leaflets*<br><br>*e.g. what time your appointment is, how to get there, what happens if the computer isn't working – how long is the wait until the next appointment etc?*<br><br>*e.g. leaflet, prison radio, peer advisors*
	**Having an appointment**	
15 mins	Unlike with outside appointments, you should know the time and date of your appointment in advance. Hopefully this will allow you to prepare any questions or worries you want to ask the hospital doctor. So let's imagine it's the day of your appointment<br><br>**Can you imagine what it will be like to participate in your appointment?**	*e.g. hard to get to healthcare, hard to prepare questions, hard to*

IRAS study number: 229646, Version 1.1, 03/10/19

	**How do you think you would feel during your video appointment?**	*engage with doctor over internet*<br><br>*e.g. confidence, that you can engage well and explain yourself over video link, that you would be understood*
	**Wrap up and close**	
15 mins	**Is there anything else you want to add about video appointments in prisons?**<br><br>Thanks – further follow up details etc.	

IRAS study number: 229646, Version 1.1, 03/10/19
