# Supplementary material for: Using telemedicine to improve access, cost and quality of secondary care for people in prison in England: a hybrid type 2 implementation effectiveness study
Source: BMJ Open. 2020 Feb 18;10(2):e035837. doi: 10.1136/bmjopen-2019-035837 (PMC7044812; doi:10.1136/bmjopen-2019-035837)
Supplement: Author's manuscript [file bmjopen-2019-035837.draft_revisions.pdf]

BMJ Open is committed to open peer review. As part of this commitment we make the peer review history of every article we publish publicly available.

When an article is published we post the peer reviewers' comments and the authors' responses online. We also post the versions of the paper that were used during peer review. These are the versions that the peer review comments apply to.

The versions of the paper that follow are the versions that were submitted during the peer review process. They are not the versions of record or the final published versions. They should not be cited or distributed as the published version of this manuscript.

BMJ Open is an open access journal and the full, final, typeset and author-corrected version of record of the manuscript is available on our site with no access controls, subscription charges or pay-per-view fees (http://bmjopen.bmj.com).

If you have any questions on BMJ Open's open peer review process please email
info.bmjopen@bmj.com

BMJ Open

Using Telemedicine to Improve Access, Cost, And Quality of Secondary Care for People in Prison in England: Study Protocol

Journal:	*BMJ Open*
Manuscript ID	bmjopen-2019-035837
Article Type:	Protocol
Date Submitted by the Author:	18-Nov-2019

Complete List of Authors:	Edge, Chantal; University College London, Collaborative Centre for Inclusion Health<br>George, Julie; University College London, Institute of Health Informatics<br>Black, Georgia; University College London, Applied Health Research; Dr<br>Gallagher, Michelle; Royal Surrey County Hospital NHS Foundation Trust, Gastroenterology and Hepatology<br>Ala, Aftab; Royal Surrey County Hospital NHS Foundation Trust, Gastroenterology and Hepatology<br>Patel, Shamir; Central and North West London NHS Foundation Trust, Offender Care<br>Edwards, Simon; Central and North West London NHS Foundation Trust<br>Hayward, Andrew; University College London, Institute of Epidemiology and Health Care

Keywords:	Telemedicine < BIOTECHNOLOGY & BIOINFORMATICS, FORENSIC MEDICINE, Protocols & guidelines < HEALTH SERVICES ADMINISTRATION & MANAGEMENT, INTERNAL MEDICINE

SCHOLARONE™
Manuscripts

I, the Submitting Author has the right to grant and does grant on behalf of all authors of the Work (as defined in the below author licence), an exclusive licence and/or a non-exclusive licence for contributions from authors who are: i) UK Crown employees; ii) where BMJ has agreed a CC-BY licence shall apply, and/or iii) in accordance with the terms applicable for US Federal Government officers or employees acting as part of their official duties; on a worldwide, perpetual, irrevocable, royalty-free basis to BMJ Publishing Group Ltd ("BMJ") its licensees and where the relevant Journal is co-owned by BMJ to the co-owners of the Journal, to publish the Work in this journal and any other BMJ products and to exploit all rights, as set out in our licence.

The Submitting Author accepts and understands that any supply made under these terms is made by BMJ to the Submitting Author unless you are acting as an employee on behalf of your employer or a postgraduate student of an affiliated institution which is paying any applicable article publishing charge ("APC") for Open Access articles. Where the Submitting Author wishes to make the Work available on an Open Access basis (and intends to pay the relevant APC), the terms of reuse of such Open Access shall be governed by a Creative Commons licence – details of these licences and which Creative Commons licence will apply to this Work are set out in our licence referred to above.

Other than as permitted in any relevant BMJ Author's Self Archiving Policies, I confirm this Work has not been accepted for publication elsewhere, is not being considered for publication elsewhere and does not duplicate material already published. I confirm all authors consent to publication of this Work and authorise the granting of this licence.

For peer review only

Title: Using Telemedicine to Improve Access, Cost, And Quality of Secondary Care for People in Prison in England: Study Protocol

First author (and corresponding author): Chantal Edge, UCL Collaborative Centre for Inclusion Health

Correspondence details: c.edge@ucl.ac.uk

Postal Address: Room 350, 1-19 Torrington Place, London WC1E 7HB, UK

Co-authors:

- Dr Julie George, UCL Institute of Health Informatics
- Dr Georgia Black, UCL Department of Applied Health Research
- Dr Michelle Gallagher, Department of Gastroenterology and Hepatology, Royal Surrey County Hospital NHS Foundation Trust
- Aftab Ala, Clinical Director Gastroenterology and Hepatology, Royal Surrey County Hospital NHS Foundation Trust; Department of Clinical and Experimental Medicine, University of Surrey; Institute of Liver Studies, King's College Hospital NHS Foundation Trust
- Dr Shamir Patel, Clinical Director of Offender Care, Central and North West London NHS Foundation Trust
- Dr Simon Edwards, Medical Director, Central and North West London NHS Foundation Trust
- Dr Andrew Hayward, UCL Institute of Epidemiology and Health Care

Abstract

Introduction

People in prison tend to experience poorer health, access to health care services and health outcomes than the general population. Use of video consultations (telemedicine) has been proven effective at improving the access, cost and quality of secondary care for prisoners in the USA and Australia. Implementation and use in English prison settings has been limited to date despite political drivers for change.

We plan to research the implementation of a new prison-hospital telemedicine model in an English county to understand what factors drive or hinder implementation, and whether the model can improve healthcare outcomes as demonstrated in other contextual settings.

Methods and analysis

We will undertake a hybrid type 2 implementation effectiveness study to gather evidence on both clinical and implementation outcomes. Data collection will be guided by the theoretical constructs of Normalisation Process Theory. We will prospectively collect data through: 1) prisoner/patient focus groups, interviews and questionnaires, 2) prison healthcare, hospital and wider prison staff interviews and questionnaires, 3) Routine quality improvement and service evaluation data.

For peer review only

Prison-hospital telemedicine represents a complex intervention, spanning multiple providers (hospital community, primary care and prison) within the context of the prison system and a rapidly shifting community healthcare and commissioning setting. Robust research is required to understand why implementation fails or succeeds in this context.

Undertaking research in prisons is itself also complex. To our knowledge only a handful of implementation studies have been proposed in criminal justice settings worldwide. The evidence generated from this study will be used to inform wider telemedicine implementation if outcomes suggest both the intervention and implementation can be achieved successfully in the English context.

Ethics and dissemination

This study has received NHS REC, HMPPS NRC and HRA approval. Dissemination of results will take place through peer reviewed journals, conferences and existing health and justice networks.

Article Summary

Strengths and limitations of this study

- This research is studying the real-time implementation of a complex digital intervention within prison settings.
- Peer researchers from the Prison Reform Trust are helping to deliver PPI and qualitative data collection in prisons, likely to improve patient disclosure and research engagement.
- This telemedicine intervention is not planned for delivery in any high security (Category A) prisons, therefore those who may experience the most problems accessing hospitals are not included in the patient post-telemedicine sample.

Word count: 4219

Keywords: prison, telemedicine, secondary care

Protocol Version: 1

Introduction

Prisoners tend to have poorer access to secondary healthcare than people living in the community, despite numerous national and international directives which cite the right of prisoners to equivalence of health care.(1, 2) Access to healthcare in prisons must be operationalized within the security constraints of the prison environment. All prisons in England have primary care services on site; however, secondary care services will normally be accessed off-site at local hospitals, with prisoners

For peer review only - http://bmjopen.bmj.com/site/about/guidelines.xhtml

escorted to these premises by prison officers, at cost to the National Health Service (NHS).((3) Given the resource requirement for off-site transfer and the high burden of disease experienced by people in prison, it is not unusual for patients to experience lengthy waits for non-urgent care before commencing treatment. If the need for an emergency appointment arises on the day, this will likely take precedence over the scheduled outpatient transfer. Patients may also be reluctant to attend off-site appointments due to the stigma they anticipate and experience in handcuffs at local hospital sites.(4) In addition, due to concerns that prisoners may make plans to abscond, prisoners are not permitted to know the time and date of their off-site appointment, meaning they wait for an undetermined period of time with anxiety after referral.

One approach which has been used to address these issues is telemedicine. Telemedicine refers to the use of technology, including video link, to allow remote consultation for patients without the need for direct physical contact with local health services. Telemedicine consultations have been used in prisons worldwide to reduce inequities in healthcare access experienced by prisoners(5-11), most notably in the USA and Australia.(12) There is a large body of evidence which demonstrates their effectiveness at improving the access and cost of healthcare provision in prisons.(13) The more limited data on patient experience suggests it also offers improved care quality for prisoners.(14-17)

The English prison system has not yet succeeded in implementing telemedicine at scale despite several previous attempts. (18-20) No current evidence exists thus far to describe why these models did not succeed and whether the lack of success was due to ineffective implementation or failure of the intervention itself. There is political impetus to introduce video consultations in England both in general secondary care sites as part of the NHS Long Term Plan digital agenda,(21) and within prisons due to the anticipated improvements in both access to care and cost effectiveness demonstrated by individual models elsewhere.(22-25)

Surrey is an English County (population 1.185 million)(26) housing approximately 2600 prisoners within five prisons, totalling 3% of the total prisoner population of England and Wales.(27). Surrey is commencing implementation of a local prison-hospital telemedicine model as a new way of delivering clinical care to prisoners. At the time of writing this protocol, community healthcare services (defined as health care provided outside prisons) are in a period of complex change as they seek to introduce new integrated healthcare delivery partnerships called Integrated Care Systems (ICSs).(28) The Surrey Heartlands ICS is looking to radically reduce face to face outpatient appointments with telemedicine likely to be one method deployed to meet this aim, and therefore prisons are acting as an early test bed for this intervention. Services deployed to prisons will focus on specific clinical areas at outset, namely hepatology, gastroenterology and sexual health. The telemedicine intervention and the care pathways designed for use will remain the responsibility of the NHS as part of standard clinical care. Her Majesty's Prison and Probation Service (HMPPS) is supportive of telemedicine implementation provided staff comply with several conditions surrounding its usage including the presence of a prison healthcare staff member during a telemedicine appointment alongside the patient.

For peer review only - http://bmjopen.bmj.com/site/about/guidelines.xhtml

Within this research study we will seek to understand whether telemedicine implementation between English prisons and hospitals is feasible and acceptable (to service providers, frontline staff and patients), the perceived barriers to overcome, and whether it produces the range of improved outcomes (e.g. access) hypothesised by providers. Through our extensive engagement with senior stakeholders in the Health and Justice system in England this model of evidence will be used to understand whether wider implementation of prison-hospital telemedicine should be attempted in other English regions, and if so, which elements comprise an effective implementation strategy.

Methods and analysis

Study Design

We will undertake a hybrid type 2 implementation-effectiveness study(29) to study the implementation of prison-hospital telemedicine in England. Hybrid type 2 studies have both a primary implementation outcome and a primary clinical outcome, and aim to generate evidence of a proven clinically effective intervention (prison telemedicine) on patient outcomes (access to secondary care) within a new context (English prisons).

The primary implementation outcome is the acceptability and feasibility of prison–hospital telemedicine in this context, and will be measured at different staff levels from different providers and for patients. The primary clinical effectiveness outcome is access to secondary care for people in prison in England, with secondary outcomes referring to the quality and cost of hospital care using this intervention.

Setting

Up to four prisons and three hospital settings in Surrey (England) will be included in the research, dependent on their telemedicine readiness during the study period. It is not possible to determine how many consultations will take place during the study period as hospital appointment numbers for prisoners are traditionally lower than the need within this population, given the barriers to accessing hospital care from prisons. The four prisons proposed include male and female prisoners, remand (not yet sentenced) and sentenced individuals, and different security categorisations.

Participants

Staff

There are two types of clinical provider staff who will be sampled in this project.

1. Prison healthcare staff who act as a contracted service within the Surrey prisons, but who are primarily employed by a central London NHS Foundation Trust. These staff will be responsible for referring patients to local community hospitals from prisons, local implementation and operation of the telemedicine system in prisons, attendance in telemedicine consultations with patients and liaison with prison staff for arrangement of patient transportation.

For peer review only - http://bmjopen.bmj.com/site/about/guidelines.xhtml

2. Hospital staff employed within Surrey community hospitals who will be delivering secondary care to patients in prisons over the telemedicine link. There are two Surrey hospitals who may be sampled for this research dependant on telemedicine readiness. Pre-implementation interviews will select staff from across all organisations. Throughout this protocol we refer to these groups as: prison healthcare staff, hospital staff or all staff (prison and community hospital staff combined).

Wider prison staff from participating prisons will also take part as research participants. This will reflect their role in scheduling patient offsite transfers and escorting patients to the prison healthcare department for telemedicine appointment.

Staff sampling

Participants for interviews prior to and during implementation will be selected using a combination of purposive and snowball sampling to generate a key informant sample. Interviewees will be selected from across all departments/prisons participating and will comprise a mix of staff from across macro (board level), meso (departmental lead/managers) and micro levels (frontline delivery of telemedicine).

At the end of the telemedicine data collection period, semi-structured 1:1 interviews will be conducted with key staff informants from all clinical staff and wider prison staff (e.g. prison officers). Interviews will be split amongst a) staff who have experience of using/contributing to others use of telemedicine and b) staff from wider specialties who may wish to use telemedicine for prison consultations in the future. Purposive and snowball sampling will be used to identify staff interview participants with a strategic view of the health system who can comment on wider clinical specialties that may be appropriate for delivery via telemedicine in prisons, based on lessons learnt from implementation of the current services.

Prison staff (e.g. prison officers) from participating prisons will also be approached for participation in research activities, with participant numbers guided by the responsible prison Governor.

Prisoners

We will include prisoners from both telemedicine and telemedicine naïve prisons as participants within this study.

1. Patients in telemedicine prisons who use the system for an appointment will be invited by prison healthcare staff to complete a post appointment questionnaire and an opportunity to participate in a 1:1 interview with the research team.

2. In conjunction with the Prison Reform Trust charity (PRT) (30) we will organise and run focus groups (n=5) in five ‘telemedicine naïve’ prisons outside of the Surrey footprint (i.e. those who have no prior experience of telemedicine/ are not preparing for imminent telemedicine implementation). Participants will be recruited via existing PRT networks within the prisons.

For peer review only - http://bmjopen.bmj.com/site/about/guidelines.xhtml

For peer review only

Consent

Informed consent for participation from health care staff will be taken by the researcher. Informed consent from prisoners in focus groups will be taken by the Prison Reform Trust charity. Informed consent from prisoners in interviews will be taken by the researcher in the presence of a prison healthcare staff member.

Key Inclusion and Exclusion Criteria

Staff

For stage 1 staff interviews staff must have a stakeholder role in local telemedicine implementation and delivery. For stage 2 acceptability/feasibility interviews stuff must have either been involved in implementing telemedicine intervention in Surrey OR have used the intervention (either accompanying patients in consultations or providing secondary care treatment) OR work as a staff member in a prison offering telemedicine. For stage 2 interviews on wider telemedicine applicability staff must have either responsibility for prioritising or approving off-site appointments to hospitals for patients in prisons OR provide secondary care to offenders OR involved in healthcare delivery to prisons including strategic level staff.

Prisoners

Prisoner participation in any research activity in a prison is ultimately vetted and granted by prison staff in light of safety concerns regarding the individual. Several general exclusion criteria exist for participants in prisons which are common across all research activities proposed. These include: people who cannot give informed consent, people who are deemed a risk to researchers/not permitted to participate (by prison officials). In addition patients who move prison after their telemedicine consultation will not be approached for interview unless they have moved to another one of the study prisons where HMPPS ethical approval is valid.

To participate in patient questionnaires or patient interviews, the prisoner must have used the local telemedicine system. Participation in telemedicine focus groups will be open to all prisoners who are not excluded based on the criteria above and who currently reside in a telemedicine naïve prison.

Theoretical framework

Use of theory in implementation research both guides the collection and analysis of data and provides explanations for the phenomena observed.(31) Our implementation research will be informed primarily by Normalisation Process Theory (NPT)(32) which has been used to describe and explain implementation outcomes in numerous studies of complex healthcare interventions.(31)

Normalisation Process Theory focusses primarily on the work that individuals and groups undertake to operationalise and normalise an intervention. The theory proposes four constructs: Coherence, Cognitive Participation, Collective Action and Reflexive Monitoring.(32, 33) As identified in a systematic review of prison telemedicine,(12) staff attitude and behaviour is reported to be both the

For peer review only - http://bmjopen.bmj.com/site/about/guidelines.xhtml

main driver and barrier to successful implementation, making NPT particularly relevant to this intervention. Successful implementation elsewhere has found that staff at macro (board level), meso (departmental lead/managers) and micro levels (frontline delivery of telemedicine) must be engaged and enthused in the provision of the intervention, across a multiplicity of providers (community hospital, prison healthcare, wider prison staff) all who have differing needs, wants and beliefs. (12)

Most proposed evaluation activities will focus on staff perceptions of feasibility and acceptability. Data collection (e.g. interview topic guides) will be guided by the constructs of NPT and research findings will be reported using a mixed methods approach under NPT construct headings.

Normalisation Process Theory calls for a description of the intervention and the context in which it will be deployed. Through interviews and use of the NOMAD survey(33) we will gather staff data on these factors. NPT has previously been criticised for its focus on individual and collective agency, and not paying enough attention to the wider organisational and relational contexts of the implementation.(31) Therefore, to provide more generalisable contextual information, in parallel to NPT, several constructs from the Consolidated Framework for Implementation Research (CFIR) will be used to guide an in-depth description of the intervention itself (core components and adaptable peripheries), a description of the outer setting (economic, political, and social context) and the inner setting (structural, political, and cultural contexts through which the implementation process will proceed).(34) In this way CFIR will be used to identify potential local contextual implementation barriers and facilitators that will be specifically explored within the individual/collective domains of NPT, which in itself will provide explanation as to why these matter in this context and the interrelations amongst constructs.

NPT refers to the opinions and reflections of staff involved in the intervention under study and the work they undertake to deliver the intervention. In the general community, patients are also required to undertake 'work' to access healthcare appointments. For example, booking the appointment, attending the appointment and remembering to take their medication. However, given the limited autonomy of patients in prison their ability to perform this 'work' is restricted. This means that NPT does not necessarily form the best framework for understanding prisoner data, despite the fact that their views on telemedicine will indicate whether it has a chance of normalisation (i.e. use). Prisoners will always be able to opt for standard care as usual instead of telemedicine. For this reason, prisoner data will be analysed using the Theoretical Framework of Acceptability (TFA) (35) which can be used to access prospective and retrospective acceptability of interventions to recipients where autonomy may be constrained. TFA constructs include: Affective Attitude, Burden, Ethicality, Intervention Coherence, Opportunity Costs, Perceived Effectiveness and Self-Efficacy. These will be used to guide both pre and post implementation data collection with patients.

Patient and Public Involvement

Two PPI representatives with lived experience of imprisonment assisted with preparation of study materials prior to ethical submissions and attend steering group meetings in the community. Following NRC approval PPI groups are being convened within prisons (1 male, 1 female) to input into ongoing

For peer review only - http://bmjopen.bmj.com/site/about/guidelines.xhtml

research activities e.g. patient questionnaire design. These groups will each meet three times during the study, with continuity of membership if possible within the prison environment.

Research Activities and Quality Improvement measures

Research activities planned are shown in Figure 1. Several quality improvement measures will run in parallel to research activities and complement the final analysis.

Figure 1

Pre-implementation Research Activities (RAs)

RA1: Staff Interviews (Stage 1)

1:1 semi-structured interviews (maximum n=30) amongst telemedicine stakeholders from all staff groups to understand anticipated benefits, barriers and enablers to telemedicine usage in a prison-hospital setting, within the English commissioning context. The interview guide has been developed based on the NPT constructs.

RA2: NOMAD questionnaire

The NOMAD questionnaire is an existing validated questionnaire, based on the principles of normalisation process theory (Finch et al., 2018). The NOMAD questionnaire seeks to identify barriers to normalisation of an intervention in healthcare to explain why interventions fail to embed in day to day use. We will administer this anonymous email survey questionnaire to all healthcare staff and additionally to prison officers at participating prisons. This will collect data on staff perceptions and concerns surrounding telemedicine. This questionnaire will be administered prior to telemedicine implementation and several months after implementation, and the changes in response quantified.

RA5: Prison Focus Groups

Study prisons have been advocating for telemedicine introduction for several years and prisoners are aware it may soon be introduced. We will collect data to represent broader prisoner views on telemedicine in prison focus groups (n=5) where there is currently no 'threat' of implementation and prisoners will be free to speak openly of their concerns. Results will be used where applicable to inform the current telemedicine service implementation and to inform policy for future telemedicine services if research results suggest it can be widely implemented. For example: "What do you think would stop people wanting to use a telemedicine system and how could we mitigate this?" The topic guide for focus groups will be guided by the TFA and PPI groups.

For peer review only - http://bmjopen.bmj.com/site/about/guidelines.xhtml

Implementation Research Activities

These activities will commence after telemedicine has become operational in at least one prison site.

RA1: Staff Interviews (Stage 1) continued

Frontline delivery staff will be re-interviewed during implementation to understand how the telemedicine services proposed at outset have changed and adapted as issues arise, and what specific barriers have arisen that were not anticipated.

RA3: Clinician questionnaire on telemedicine appointment

Both hospital clinicians and prison healthcare staff involved in a telemedicine appointment will complete a paper questionnaire at the close of each appointment. This will seek to understand whether the appointment was clinically acceptable, with reference to anticipated barriers identified in the staff interviews at outset.

The questionnaire will be refined after clinical staff interviews have been conducted. We currently anticipate the questions shown in Figure 2.

Figure 2

RA4: Patient questionnaire on telemedicine appointment

All patients who have a telemedicine appointment will be offered a short, anonymous paper questionnaire immediately after their appointment finishes, to collect data on patient acceptability of telemedicine appointments. Questionnaires should provide information that may be overlooked as 'not important' or not recalled in later patient interviews e.g. *"Did the doctor speak over you?"*

The content of the questionnaire will be based on the Telemedicine Usability Questionnaire (TUQ)(36) with several additional questions pertaining to appointments in prison. For example, *"Were you able to get to your appointment on time?"* which refers to the fact that prison officers will need to escort patients to their telemedicine appointment within the prison healthcare department.

The questionnaire will be finalised with input from PPI groups.

RA6: Patient 1:1 interviews

1:1 semi-structured interviews (n=15) will be held with patients who have had a telemedicine consultation to collect information on patient experience and acceptability. Where applicable participants will be asked to compare telemedicine treatment to previous offsite secondary care appointments.

RA7: Staff interviews (stage 2)

Interviews with staff who have used the telemedicine system with seek to understand the feasibility and acceptability of the current delivery model. Interview topic guides will be informed by NPT constructs and results of the NOMAD survey previously administered.

For peer review only - http://bmjopen.bmj.com/site/about/guidelines.xhtml

Quality Improvement (QI) Measures

The NHS Friends and Family Test

Alongside the research activities a standard NHS QI tool (the Friends and Family test) will be administered by the prison healthcare staff to patients, to monitor satisfaction of telemedicine services for all appointments. Anonymised data from this will be provided to the research team to supplement the research summary and will be compared to pre-telemedicine satisfaction scores.

Quantitative Service Evaluation - Pre and Post Anonymised Health Records Data for Specialties under Study

After nine months of service delivery routine service evaluation data will be used to assess clinical effectiveness, specifically to understand whether appointments happened more quickly using telemedicine and whether cancellations were reduced. This data will be collected and analysed by the NHS as part of their routine clinical evaluation of a service change. Aggregate anonymised electronic health records data will be provided to the research team to supplement the research summary including: number of secondary care consultations and referrals, time from referral to treatment, total number of off-site transfers, number of and reason/s for appointment cancellation. This data will be compared at stages throughout implementation to pre-telemedicine data on the same indicators, to account for a transition phase as the service becomes established.

Service Evaluation to Inform Wider Specialties for Telemedicine Delivery

To understand which other specialties may be a priority for prison telemedicine delivery aggregate, anonymised prison healthcare records data will be provided to the research team. This will provide information on the need and distribution of all secondary care outpatient appointments for people in participating prisons including: hospital and specialty referred to e.g. cardiology, time between referral and appointment by specialty, cost of prison officer escorts for appointment and cancellation data (if applicable), i.e. reason for cancellation and date of cancellation by specialty.

This analysis will help inform which other hospital specialties may be areas of value for telemedicine development e.g. department with most referrals, department with longest wait, department with most cancellations. This will complement stage 2 staff interviews on wider telemedicine specialties.

Economic analysis

Cost modelling will be undertaken by a health economist to understand how the wider rollout of prison telemedicine across specialties may affect cost effectiveness of secondary care delivery to prisoners (including costs to prison healthcare provider, wider prison costs and NHS costs). Data on resource associated with delivery of telemedicine (e.g. staff time and processes) will be collected in staff feasibility/acceptability stage 2 interviews. Individual prisons, the prison healthcare provider and community hospitals will provide costings data associated with the delivery of the telemedicine model compared with standard care which will be attributed to reported data.

For peer review only - http://bmjopen.bmj.com/site/about/guidelines.xhtml

Confidentiality

Qualitative data will be anonymised in published reports and upon archiving at the close of the study to protect participant confidentiality. Interview/focus group data will be recorded on an encrypted dictaphone. Service evaluation data will be provided as aggregate, anonymised data to remove the risk of deductive disclosure. Questionnaires from both staff and patients will be anonymous.

Ethics and Dissemination

This study has received ethical approval from the London South East NHS Research Ethics Committee (19/LO/0098), the HMPPS National Research Committee (NRC 2018-212) and Health Research Authority and Health and Care Research Wales (HRA IRAS 229646).

Study results will be published in peer reviewed journals and presented at academic conferences. Wider dissemination will be achieved through health and justice networks/organisations such as Public Health England, The Prison Reform Trust and the Royal College of General Practitioners Secure Environment Group.

Discussion

The potential for the prison context to restrict research activities should not be underestimated. Undertaking research in prisons is complex given the restrictive nature of the environment and the need to operate within the prison regime, which will inevitably differ by establishment. Health research approvals must be granted by the research sponsor, an NHS research ethics committee, the Health Research Authority (who provide general health service agreement) and individual NHS hospitals. In addition, for research taking place in prisons the HMPPS National Research Committee must provide approval which, alongside methodological scrutiny, they grant in line with their guidance:

"All research must be of benefit to HMPPS and the links to HMPPS priorities must be explicit."(37)

Finally individual Prison Governors must provide agreement that research can take place within their given establishment.

Data collection is also more challenging than in community settings. Prison electronic health records currently sit separately from community NHS records and are jointly controlled by both prison healthcare provider and NHS prison commissioners. Data collection direct from prisoners is reliant on the ability to access patients. When using questionnaires these must account for a population which is likely to have low literacy levels and an element of mistrust in research. In addition, as identified in our forthcoming literature review,(12) prison and hospital healthcare providers are likely to anticipate and experience different and unequal benefits, barriers and facilitators which may affect enthusiasm for implementation.

To undertake this research, the Principal Investigator (PI) is assuming the role of an embedded researcher, which is known to present challenges to rigorous research within healthcare organisations.(38) In this instance the PI will be embedded both within the community healthcare

For peer review only - http://bmjopen.bmj.com/site/about/guidelines.xhtml

team/s and the prison healthcare team alongside their academic base. This may compromise their ability to truly embrace 'dual' affiliation and relationship building given that relationships must be formed with two different hospital provider teams.

In summary, a telemedicine model straddling community and prison healthcare providers, situated within the context of the English prison system represents a highly complex intervention. For this reason, we have determined it appropriate to only study prisons within one county of England, which houses both male and female prisons of different security categorisation. This may restrict our sample size; however, attempts to widen the study to other counties would introduce other prisons, other hospitals, other providers and other commissioners and leave the study prone to failure as we strive to understand whether successful implementation is possible.

Potential Significance of Research

If established effectively, prison-hospital telemedicine offers the potential to improve access and quality of secondary care for prisoners, and to reduce NHS costs by reducing the amount of offsite escorts from prison to hospital that must be reimbursed from public NHS funds. There is an opportunity cost to all NHS spending, with one funded service or treatment representing an opportunity foregone to invest elsewhere.

Prisoners experience a disproportionately higher burden of disease than people within the general community including infectious diseases, long-term conditions and mental health problems.(39) The prison population is typically characterised as younger but the number of older prisoners continues to rise, bringing new challenges in the form of treatment of multiple co-morbidities within prison environments.(40) Telemedicine may be one approach to improve the health of people in prisons, which can in turn bring community dividends such as reducing reoffending and a reduction in the wider reservoir of infectious disease.(41, 42) However, it cannot hope to bring these benefits for a traditionally under-served and under-researched population, unless the implementation is successful.

In addition, the study of implementation science within criminal justice settings is highly limited. At the time of writing this protocol, we have identified only three articles pertaining to implementation research in health and justice settings.(43-45) This study will therefore contribute knowledge to an emergent and complex field of study.

Declarations

Ethics approvals

This study has received ethical approval from the London South East NHS Research Ethics Committee (19/LO/0098), the HMPPS National Research Committee (NRC 2018-212) and Health Research Authority and Health and Care Research Wales (HRA IRAS 229646).

For peer review only - http://bmjopen.bmj.com/site/about/guidelines.xhtml

Study Sponsor

University College London (Joint Research office) email: uclh.jro-communications@nhs.net

Competing interests

The authors declare no competing interests.

Funding

CE is funded by a National Institute for Health Research (NIHR), (Clinical Doctoral Research Fellowship ICA-CDRF-2017-03-006) for this research project. JG is partially funded by a Health Education England/National Institute of Health Research Clinical Lectureship (ICA-CL-2016-02-024). AH is an NIHR senior investigator. This publication presents independent research funded by the National Institute for Health Research (NIHR). The views expressed are those of the author(s) and not necessarily those of the NHS, the NIHR or the Department of Health and Social Care. The funder and sponsor has no role in study data collection, management, analysis, interpretation or writing/decisions to submit publications.

Authors' contributions

CE conceived the project. CE wrote the original research grant funding application and protocol, with methodological guidance and support from academic supervisors AH, JG and GB. SE, SP, MG, AA are senior clinicians within participating hospitals who have guided methodological considerations throughout and provide clinical supervision of the PhD student. SP and MG are named local Principle Investigators, AH is the named Chief Investigator for the study for ethical approvals. CE is the named Principle Investigator and PhD student. All authors reviewed and commented on the final draft of this protocol.

Acknowledgements

We are grateful to the University College London Joint Research Office, and the Research and Development departments at Royal Surrey County Hospital NHS Foundation Trust and Central North West London NHS Foundation Trust, for their support and assistance in developing this research study.

Authors' information (optional)

CE is undertaking this study as a PhD study which forms the basis of her NIHR Clinical Doctoral Research Fellowship. The named Chief Investigator for the study is her primary PhD supervisor AH in line with University College London study sponsorship policy.

Bibliography

1. Charles A, Draper H. 'Equivalence of care' in prison medicine: is equivalence of process the right measure of equity? J Med Ethics. 2012;38(4):215.
2. Assembly UG. Nelson Mandela Rules. United Nations; 2015.

For peer review only - http://bmjopen.bmj.com/site/about/guidelines.xhtml

3. Leaman J. Rapid review of evidence of the impact on health outcomes of NHS commissioned health services for people in secure and detained settings to inform future health interventions and prioritisation in England. In: England PH, editor. London, UK2016.
4. Association BM. The medical role in restraint and control: custodial settings. London, UK: British Medical Association; 2009.
5. Bara C, Maillard H, Beneton N, Serre P, Celerier P. Teledermatology in the management of skin diseases in prison inmates: Experience in central France. [French]. Ann Dermatol Venereol. 2013;140(3):216-8.
6. Blanchet K. Innovative programs in telemedicine: University of Texas Medical Branch at Galveston Electronic Health Network. Telemed J e-Health. 2005;11(2):116-23.
7. Braniff C, Connelly C, Mollison L. Telemedicine allows successful Hepatitis C treatment in Western Australian prisons. J Viral Hepatitis. 2015;22:90-.
8. Chipps J, Ramlall S, Mars M. Practice guidelines for videoconference-based telepsychiatry in South Africa. Afr J Psychiatry. 2012;15(4):271-82.
9. Fallon PV. Introducing telehealth to English prisons. Int J Prison Health. 2007;3(1):77-8.
10. Gualano MR, Bert F, Andriolo V, Grosso M, Minniti D, Siliquini R. Use of telemedicine in the European penitentiaries: current scenario and best practices. Eur J Public Health. 2017;27(1):30-5.
11. Lee YM. A debate about telemedicine in South Korea. J Korean Med Assoc. 2016;59(11):828-31.
12. Edge C, Black, G., King, E., George, J., Patel, S., Hayward, A. Improving care quality with prison telemedicine: the effects of context and multiplicity on successful implementation and use. The Journal of Telemedicine and Telecare. 2019 Oct 22:1357633X19869131. doi: 10.1177/1357633X19869131..
13. Wheeler J, Hinton E. Effectiveness of telehealth on correctional facility health care: A systematic review protocol. JBI Database Syst. 2017;15(5):1256-64.
14. Caudill RL, Sager Z. Institutionally based videoconferencing. Int Rev Psychiatry. 2015;27(6):496-503.
15. Garfield MJ, Watson RT. Four case studies in state-supported telemedicine initiatives. Telemed J e-Health. 2003;9(2):197-205.
16. Lyckhoml LJ, Justis D. Delivery of cancer care to inmates of correctional facilities through telemedicine. Journal of Clinical Oncology; Jul2004. p. 764S-S.
17. Magaletta PR, Fagan TJ, Peyrot MF. Telehealth in the federal bureau of prisons: Inmates' perceptions. Prof Psychol-Res Pract. 2000;31(5):497-502.
18. Salem Y, editor Forensic telepsychiatry in UK. European Psychiatry Conference: 18th European Congress of Psychiatry Munich Germany Conference Publication:; 2010.
19. Pearce L. Nurses lead the way with webcam consultations. Nurs Stand. 2017;32(2):22-3.
20. Clements S. On the challenge of providing care for prisoners. HSJ. 2012;122(6331):Suppl 10.
21. NHS. The NHS Long Term Plan. UK; 2019.
22. Leonard S. The development and evaluation of a telepsychiatry service for prisoners. J Psychiatr Ment Health Nurs. 2004;11(4):461-8.
23. Service NOM. National Security Framework Ref: NSF 6.6 SECURITY MANAGEMENT FUNCTION Management and Security of Escape List (E-List) Prisoners. 2015.
24. Stewart R. Urgent Notice - HMP Birmingham. In: Justice Mo, editor. London2018.
25. Committee PHaSC. 12th report - Prison Health. www.parliament.uk2018.
26. ONS. Population Estimates. UK: Office of National Statistics; 2017.
27. Surrey-i. Surrey Prison Population. Surrey, UK: Surrey County Council; 2019.
28. England N. Integrated Care Systems [Available from: https://www.england.nhs.uk/integratedcare/integrated-care-systems/.
29. Curran GM, Bauer M, Mittman B, Pyne JM, Stetler C. Effectiveness-implementation hybrid designs: combining elements of clinical effectiveness and implementation research to enhance public health impact. Med Care. 2012;50(3):217-26.
30. Trust PR. Prison Reform Trust Website UK2019 [Available from: http://www.prisonreformtrust.org.uk/.
31. May CR, Cummings A, Girling M, Bracher M, Mair FS, May CM, et al. Using Normalization Process Theory in feasibility studies and process evaluations of complex healthcare interventions: a systematic review. Implement Sci. 2018;13(1):80.
32. Murray E, Treweek S, Pope C, MacFarlane A, Ballini L, Dowrick C, et al. Normalisation process theory: a framework for developing, evaluating and implementing complex interventions. BMC Medicine. 2010;8(1):63.

For peer review only - http://bmjopen.bmj.com/site/about/guidelines.xhtml

33. Finch TL, Girling, M., May, C.R., Mair, F.S., Murray, E., Treweek, S., Steen, I.N., McColl, E.M., Dickinson, C., Rapley, T. NoMad: Implementation measure based on Normalization Process Theory. [Measurement instrument]. 2015.
34. Damschroder LJ, Aron DC, Keith RE, Kirsh SR, Alexander JA, Lowery JC. Fostering implementation of health services research findings into practice: a consolidated framework for advancing implementation science. Implement Sci. 2009;4(1):50.
35. Sekhon M, Cartwright M, Francis JJ. Acceptability of healthcare interventions: an overview of reviews and development of a theoretical framework. BMC Health Serv Res. 2017;17(1):88.
36. Parmanto B, Lewis AN, Jr., Graham KM, Bertolet MH. Development of the Telehealth Usability Questionnaire (TUQ). International journal of telerehabilitation. 2016;8(1):3-10.
37. HMPPS. National Research Committee – Application Guidance. In: Service HMsPaP, editor. London, England.
38. Vindrola-Padros C, Pape T, Utley M, Fulop NJ. The role of embedded research in quality improvement: a narrative review. BMJ Quality & Safety. 2017;26(1):70.
39. Aldridge RW, Story A, Hwang SW, Nordentoft M, Luchenski SA, Hartwell G, et al. Morbidity and mortality in homeless individuals, prisoners, sex workers, and individuals with substance use disorders in high-income countries: a systematic review and meta-analysis. Lancet. 2018;391(10117):241-50.
40. Sturge G. UK Prison Population Statistics: Briefing Paper House of Commons Library; 2018.
41. RCGP-SEG. Equivalence of care in Secure Environments in the UK - Position Statement. Royal College of General Practitioners Secure Environments Group; 2018.
42. O'Moore E. England PH, editor. Gov.UK2015. Available from: https://publichealthmatters.blog.gov.uk/2015/07/06/the-community-dividend-why-improving-prisoner-health-is-essential-for-public-health/.
43. Pearson M, Brand SL, Quinn C, Shaw J, Maguire M, Michie S, et al. Using realist review to inform intervention development: methodological illustration and conceptual platform for collaborative care in offender mental health. Implement Sci. 2015;10(1):134.
44. Almost J, Gifford WA, Doran D, Ogilvie L, Miller C, Rose DN, et al. Correctional nursing: a study protocol to develop an educational intervention to optimize nursing practice in a unique context. Implement Sci. 2013;8(1):71.
45. Johnson JE, Wiltsey-Stirman S, Stout R, Miller T, Zlotnick C. Implementation of evidence-based mental health practices in criminal justice settings: Implications from a Hybrid Type I cost-effectiveness trial. Implement Sci. 2015;10(1):A76.

For peer review only

For peer review only - http://bmjopen.bmj.com/site/about/guidelines.xhtml

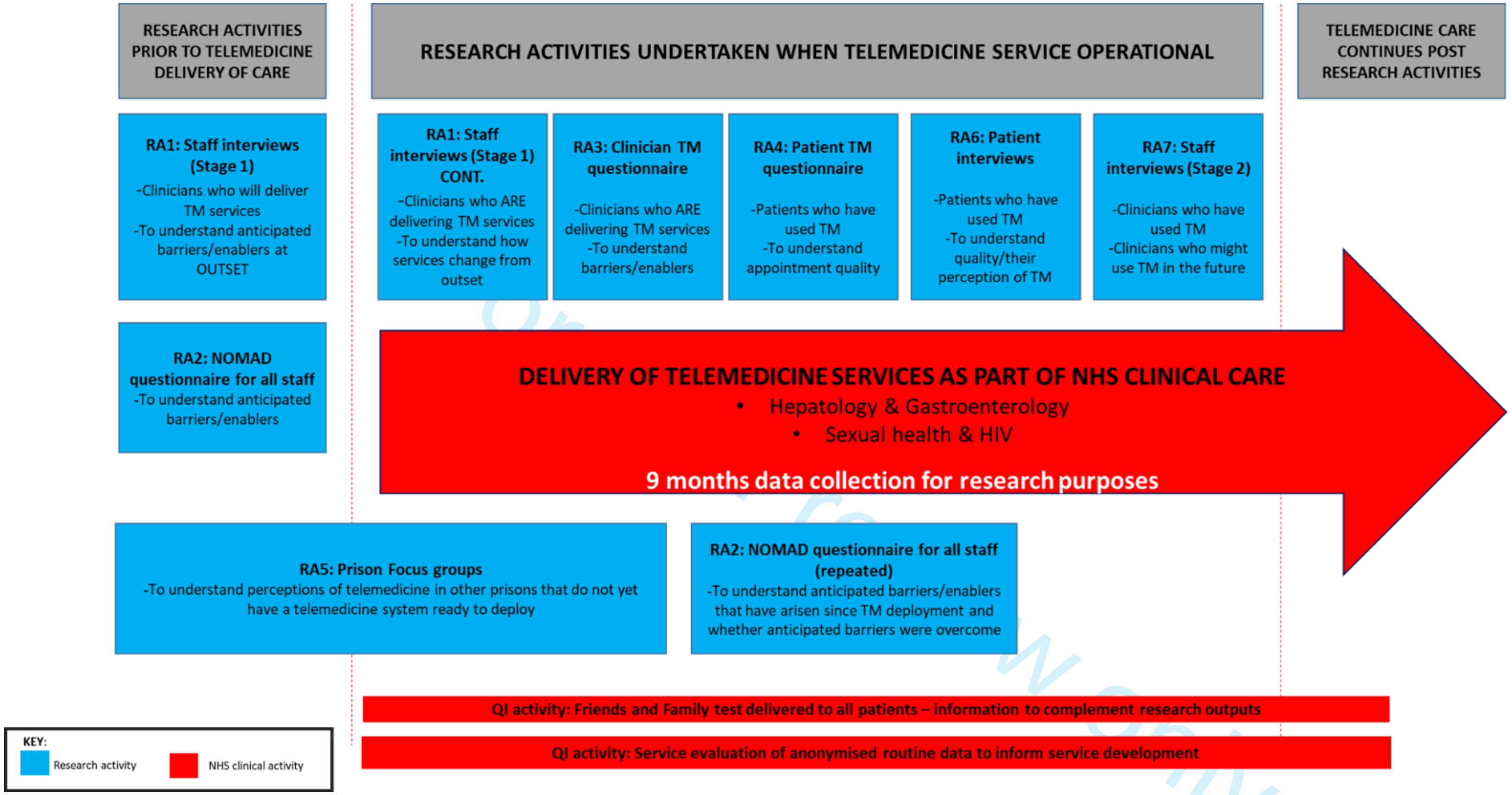

Figure 1 Study Research Activities

- Were you able to connect/log on to the system with ease?
- Was the quality of the internet connection sufficient for consultation?
- Was the telemedicine consultation sufficient to understand/treat patient clinical issues (or will an in-person appointment be required)?
- Were you able to discuss the onward treatment plan for the patient with the hospital/prison staff member?
- Would there have been difficulties in planning/delivering the patient treatment plan if you had not spoken directly with the hospital/prison clinical staff?
- Did the patient need your help to clarify any clinical information during or after the appointment?
- Did the patient appear comfortable in the telemedicine appointment?
- In hindsight, do you think the patient was appropriately referred to a telemedicine appointment? (and if not, why not?)

Figure 2 Anticipated staff questions for questionnaire post telemedicine consultation

For peer review only

Standards for Reporting Implementation Studies: the StaRI checklist for completion

The StaRI standard should be referenced as: Pinnock H, Barwick M, Carpenter C, Eldridge S, Grandes G, Griffiths CJ, Rycroft-Malone J, Meissner P, Murray E, Patel A, Sheikh A, Taylor SJC for the StaRI Group. Standards for Reporting Implementation Studies (StaRI) statement. *BMJ* 2017;356:i6795

The detailed Explanation and Elaboration document, which provides the rationale and exemplar text for all these items is: Pinnock H, Barwick M, Carpenter C, Eldridge S, Grandes G, Griffiths C, Rycroft-Malone J, Meissner P, Murray E, Patel A, Sheikh A, Taylor S, for the StaRI group. Standards for Reporting Implementation Studies (StaRI). Explanation and Elaboration document. *BMJ Open* 2017 2017;7:e013318

Notes: A key concept of the StaRI standards is the dual strands of describing, on the one hand, the implementation strategy and, on the other, the clinical, healthcare, or public health intervention that is being implemented. These strands are represented as two columns in the checklist.

The primary focus of implementation science is the implementation strategy (column 1) and the expectation is that this will always be completed.	The evidence about the impact of the intervention on the targeted population should always be considered (column 2) and either health outcomes reported or robust evidence cited to support a known beneficial effect of the intervention on the health of individuals or populations.

The StaRI standardsrefers to the broad range of study designs employed in implementation science. Authors should refer to other reporting standards for advice on reporting specific methodological features. Conversely, whilst all items are worthy of consideration, not all items will be applicable to, or feasible within every study.

Checklist item		Reported on page #	Implementation Strategy	Reported on page #	Intervention
			"Implementation strategy" refers to how the intervention was implemented		"Intervention" refers to the healthcare or public health intervention that is being implemented.
**Title and abstract**					
Title	**1**	1	Identification as an implementation study, and description of the methodology in the title and/or keywords		
Abstract	**2**	2-4	Identification as an implementation study, including a description of the implementation strategy to be tested, the evidence-based intervention being implemented, and defining the key implementation and health outcomes.		
**Introduction**					
Introduction	**3**	2-3<br>12-13	Description of the problem, challenge or deficiency in healthcare or public health that the intervention being implemented aims to address.		
Rationale	**4**	6-7	The scientific background and rationale for the implementation strategy (including any underpinning theory/framework/model, how it is expected to achieve its effects and any pilot work).	3	The scientific background and rationale for the intervention being implemented (including evidence about its effectiveness and how it is expected to achieve its effects).

For peer review only

Aims and objectives	**5**	4	The aims of the study, differentiating between implementation objectives and any intervention objectives.		
**Methods: description**					
Design	**6**	4-11	The design and key features of the evaluation, (cross referencing to any appropriate methodology reporting standards) and any changes to study protocol, with reasons		
Context	**7**	*7 (see text below)	The context in which the intervention was implemented. (Consider social, economic, policy, healthcare, organisational barriers and facilitators that might influence implementation elsewhere).		
Targeted 'sites'	**8**	4	The characteristics of the targeted 'site(s)' (e.g locations/personnel/resources etc.) for implementation and any eligibility criteria.	4	The population targeted by the intervention and any eligibility criteria.
Description	**9**	*9 (see text below	A description of the implementation strategy	*9 (see text below	A description of the intervention
Sub-groups	**10**	NA	Any sub-groups recruited for additional research tasks, and/or nested studies are described		
**Methods: evaluation**					
Outcomes	**11**	*11 (see text below	Defined pre-specified primary and other outcome(s) of the implementation strategy, and how they were assessed. Document any pre-determined targets	*11 (see text below	Defined pre-specified primary and other outcome(s) of the intervention (if assessed), and how they were assessed. Document any pre-determined targets
Process evaluation	**12**	*12 (see text below	Process evaluation objectives and outcomes related to the mechanism by which the strategy is expected to work		
Economic evaluation	**13**	11	Methods for resource use, costs, economic outcomes and analysis for the implementation strategy	11	Methods for resource use, costs, economic outcomes and analysis for the intervention
Sample size	**14**	*14	Rationale for sample sizes (including sample size calculations, budgetary constraints, practical considerations, data saturation, as appropriate)		
Analysis	**15**	10-11	Methods of analysis (with reasons for that choice)		

For peer review only

For peer review only - http://bmjopen.bmj.com/site/about/guidelines.xhtml

Sub-group analyses	**16**	NA	Any a priori sub-group analyses (e.g. between different sites in a multicentre study, different clinical or demographic populations), and sub-groups recruited to specific nested research tasks

*7 – data to provide a description of the context will be gathered through Research Activity (RA) RA1 – staff interviews
*9– full description of the intervention and implementation strategy will be described in the final write up of the research and will be informed by RA 1 & 7
*11– the outcome of the implementation strategy and intervention will be described in the final write up of the research and will be informed by RA 2-7 and service evaluation/NHS friends and family test data
*12 – process evaluation will be informed by RA1 (logic model development), RA2-7 (process measures)
*14 – sample size will be informed by the study as an aspect of feasibility

**Results**					
Characteristics	**17**	*17	Proportion recruited and characteristics of the recipient population for the implementation strategy	*17	Proportion recruited and characteristics (if appropriate) of the recipient population for the intervention
Outcomes	**18**	As *11	Primary and other outcome(s) of the implementation strategy	As *11	Primary and other outcome(s) of the Intervention (if assessed)
Process outcomes	**19**	As *12	Process data related to the implementation strategy mapped to the mechanism by which the strategy is expected to work		
Economic evaluation	**20**	As described pg 11	Resource use, costs, economic outcomes and analysis for the implementation strategy	As described pg 11	Resource use, costs, economic outcomes and analysis for the intervention
Sub-group analyses	**21**	NA	Representativeness and outcomes of subgroups including those recruited to specific research tasks		
Fidelity/ adaptation	**22**	*22	Fidelity to implementation strategy as planned and adaptation to suit context and preferences	*22	Fidelity to delivering the core components of intervention (where measured)

For peer review only - http://bmjopen.bmj.com/site/about/guidelines.xhtml

Contextual changes	**23**	*23	Contextual changes (if any) which may have affected outcomes		
Harms	**24**	*24	All important harms or unintended effects in each group		
**Discussion**					
Structured discussion	**25**	NA at protocol stage	Summary of findings, strengths and limitations, comparisons with other studies, conclusions and implications		
Implications	**26**	NA at protocol stage	Discussion of policy, practice and/or research implications of the implementation strategy (specifically including scalability)	NA at protocol stage	Discussion of policy, practice and/or research implications of the intervention (specifically including sustainability)
**General**					
Statements	**27**	14-15	Include statement(s) on regulatory approvals (including, as appropriate, ethical approval, confidential use of routine data, governance approval), trial/study registration (availability of protocol), funding and conflicts of interest		

*17 – participant demographics will be collected for all RA (including participation in the intervention via RA 4 and reported
*22 – Fidelity and adaptation will be described through comparison of RA1, RA1 cont. and RA7, and through RA2
*23 Contextual changes will be described through data collection in RA1 and RA7, and RA2
*24 Harms/unintended effects data will be collected in RA3, RA4, RA6, RA7

For peer review only

BMJ Open

Using Telemedicine to Improve Access, Cost, And Quality of Secondary Care for People in Prison in England: A Hybrid Type 2 Implementation Effectiveness Study

Journal:	*BMJ Open*
Manuscript ID	bmjopen-2019-035837.R1
Article Type:	Protocol
Date Submitted by the Author:	15-Jan-2020
Complete List of Authors:	Edge, Chantal; University College London, Collaborative Centre for Inclusion Health<br>George, Julie; University College London, Institute of Health Informatics<br>Black, Georgia; University College London, Applied Health Research; Dr<br>Gallagher, Michelle; Royal Surrey County Hospital NHS Foundation Trust, Gastroenterology and Hepatology<br>Ala, Aftab; Royal Surrey County Hospital NHS Foundation Trust, Gastroenterology and Hepatology<br>Patel, Shamir; Central and North West London NHS Foundation Trust, Offender Care<br>Edwards, Simon; Central and North West London NHS Foundation Trust<br>Hayward, Andrew; University College London, Institute of Epidemiology and Health Care
**Primary Subject Heading**:	Health services research
Secondary Subject Heading:	Gastroenterology and hepatology, Public health, Qualitative research
Keywords:	Telemedicine < BIOTECHNOLOGY & BIOINFORMATICS, FORENSIC MEDICINE, Protocols & guidelines < HEALTH SERVICES ADMINISTRATION & MANAGEMENT, INTERNAL MEDICINE

SCHOLARONE™
Manuscripts

I, the Submitting Author has the right to grant and does grant on behalf of all authors of the Work (as defined in the below author licence), an exclusive licence and/or a non-exclusive licence for contributions from authors who are: i) UK Crown employees; ii) where BMJ has agreed a CC-BY licence shall apply, and/or iii) in accordance with the terms applicable for US Federal Government officers or employees acting as part of their official duties; on a worldwide, perpetual, irrevocable, royalty-free basis to BMJ Publishing Group Ltd ("BMJ") its licensees and where the relevant Journal is co-owned by BMJ to the co-owners of the Journal, to publish the Work in this journal and any other BMJ products and to exploit all rights, as set out in our licence.

The Submitting Author accepts and understands that any supply made under these terms is made by BMJ to the Submitting Author unless you are acting as an employee on behalf of your employer or a postgraduate student of an affiliated institution which is paying any applicable article publishing charge ("APC") for Open Access articles. Where the Submitting Author wishes to make the Work available on an Open Access basis (and intends to pay the relevant APC), the terms of reuse of such Open Access shall be governed by a Creative Commons licence – details of these licences and which Creative Commons licence will apply to this Work are set out in our licence referred to above.

Other than as permitted in any relevant BMJ Author's Self Archiving Policies, I confirm this Work has not been accepted for publication elsewhere, is not being considered for publication elsewhere and does not duplicate material already published. I confirm all authors consent to publication of this Work and authorise the granting of this licence.

For peer review only

Title: Using Telemedicine to Improve Access, Cost, And Quality of Secondary Care for People in Prison in England: A Hybrid Type 2 Implementation Effectiveness Study

First author (and corresponding author): Chantal Edge, UCL Collaborative Centre for Inclusion Health

Correspondence details: c.edge@ucl.ac.uk

Postal Address: Room 350, 1-19 Torrington Place, London WC1E 7HB, UK

Co-authors:

- Dr Julie George, UCL Institute of Health Informatics
- Dr Georgia Black, UCL Department of Applied Health Research
- Dr Michelle Gallagher, Department of Gastroenterology and Hepatology, Royal Surrey County Hospital NHS Foundation Trust
- Professor Aftab Ala, Clinical Director Gastroenterology and Hepatology, Royal Surrey County Hospital NHS Foundation Trust; Department of Clinical and Experimental Medicine, University of Surrey; Institute of Liver Studies, King's College Hospital NHS Foundation Trust
- Dr Shamir Patel, Clinical Director of Offender Care, Central and North West London NHS Foundation Trust
- Dr Simon Edwards, Medical Director, Central and North West London NHS Foundation Trust
- Professor Andrew Hayward, UCL Institute of Epidemiology and Health Care

Abstract

Introduction

People in prison tend to experience poorer health, access to health care services and health outcomes than the general population. Use of video consultations (telemedicine) has been proven effective at improving the access, cost and quality of secondary care for prisoners in the USA and Australia. Implementation and use in English prison settings has been limited to date despite political drivers for change.

We plan to research the implementation of a new prison-hospital telemedicine model in an English county to understand what factors drive or hinder implementation, and whether the model can improve healthcare outcomes as demonstrated in other contextual settings.

Methods and analysis

We will undertake a hybrid type 2 implementation effectiveness study to gather evidence on both clinical and implementation outcomes. Data collection will be guided by the theoretical constructs of Normalisation Process Theory. We will prospectively collect data through: 1) prisoner/patient focus groups, interviews and questionnaires, 2) prison healthcare, hospital and wider prison staff interviews and questionnaires, 3) Routine quality improvement and service evaluation data.

For peer review only

For peer review only - http://bmjopen.bmj.com/site/about/guidelines.xhtml

Up to four prisons and three hospital settings in Surrey (England) will be included in the telemedicine research, dependent on their telemedicine readiness during the study period. Prisons proposed include male and female prisoners, remand (not yet sentenced) and sentenced individuals, and different security categorisations.

In addition, focus groups in five telemedicine naïve prisons will provide information on patient pre-conceptions and concerns surrounding telemedicine.

Ethics and dissemination

This study has received NHS REC, HMPPS NRC and HRA approval. Dissemination of results will take place through peer reviewed journals, conferences and existing health and justice networks.

Article Summary

Strengths and limitations of this study

- This research is studying the real-time implementation of a complex digital intervention within prison settings.
- Peer researchers from the Prison Reform Trust are helping to deliver PPI and qualitative data collection in prisons, likely to improve patient disclosure and research engagement.
- This telemedicine intervention is not planned for delivery in any high security (Category A) prisons, therefore those who may experience the most problems accessing hospitals are not included in the patient post-telemedicine sample.

Word count: 5280

Keywords: prison, telemedicine, secondary care

Protocol Version: 1

Introduction

Prisoners tend to have poorer access to secondary healthcare than people living in the community, despite numerous national and international directives which cite the right of prisoners to equivalence of health care.(1, 2) Access to healthcare in prisons must be operationalized within the security constraints of the prison environment. All prisons in England have primary care services on site; however, secondary care services will normally be accessed off-site at local hospitals, with prisoners escorted to these premises by prison officers, at cost to the National Health Service (NHS).((3) Given the resource requirement for off-site transfer and the high burden of disease experienced by people in prison, it is not unusual for patients to experience lengthy waits for non-urgent care before commencing treatment. If the need for an emergency appointment arises on the day, this will likely take precedence over the scheduled outpatient transfer. Patients may also be reluctant to attend off-

For peer review only - http://bmjopen.bmj.com/site/about/guidelines.xhtml

site appointments due to the stigma they anticipate and experience in handcuffs at local hospital sites.(4) In addition, due to concerns that prisoners may make plans to abscond, prisoners are not permitted to know the time and date of their off-site appointment, meaning they wait for an undetermined period of time with anxiety after referral.

One approach which has been used to address these issues is telemedicine. Telemedicine refers to the use of technology, including video link, to allow remote consultation for patients without the need for direct physical contact with local health services. Telemedicine consultations have been used in prisons worldwide to reduce inequities in healthcare access experienced by prisoners(5-11), most notably in the USA and Australia.(12) There is a large body of evidence which demonstrates their effectiveness at improving the access and cost of healthcare provision in prisons.(13) The more limited data on patient experience suggests it also offers improved care quality for prisoners.(14-17)

The English prison system has not yet succeeded in implementing telemedicine at scale despite several previous attempts. (18-20) No current evidence exists thus far to describe why these models did not succeed and whether the lack of success was due to ineffective implementation or failure of the intervention itself. There is political impetus to introduce video consultations in England both in general secondary care sites as part of the NHS Long Term Plan digital agenda,(21) and within prisons due to the anticipated improvements in both access to care and cost effectiveness demonstrated by individual models elsewhere.(22-25)

Surrey is an English County (population 1.185 million)(26) housing approximately 2600 prisoners within five prisons, totalling 3% of the total prisoner population of England and Wales.(27). Surrey is commencing implementation of a local prison-hospital telemedicine model as a new way of delivering clinical care to prisoners. At the time of writing this protocol, community healthcare services (defined as health care provided outside prisons) are in a period of complex change as they seek to introduce new integrated healthcare delivery partnerships called Integrated Care Systems (ICSs).(28) The Surrey Heartlands ICS is looking to radically reduce face to face outpatient appointments with telemedicine likely to be one method deployed to meet this aim, and therefore prisons are acting as an early test bed for this intervention. Services deployed to prisons will focus on specific clinical areas at outset, namely hepatology, gastroenterology and sexual health. Sexual health services provided will initially focus on those provided by a health advisor role, for example, counselling regarding a sexual health diagnosis or contact tracing. Hepatology and gastroenterology consultations will include routine follow ups and treatment of hepatitis C and B. The telemedicine intervention and the care pathways designed for use will remain the responsibility of the NHS as part of standard clinical care. Her Majesty's Prison and Probation Service (HMPPS) is supportive of telemedicine implementation provided staff comply with several conditions surrounding its usage including the presence of a prison healthcare staff member during a telemedicine appointment alongside the patient.

Within this research study we will seek to understand whether telemedicine implementation between English prisons and hospitals is feasible and acceptable (to service providers, frontline staff and patients), the perceived barriers to overcome, and whether it produces the range of improved

For peer review only - http://bmjopen.bmj.com/site/about/guidelines.xhtml

outcomes (e.g. access) hypothesised by providers. Through our extensive engagement with senior stakeholders in the Health and Justice system in England this model of evidence will be used to understand whether wider implementation of prison-hospital telemedicine should be attempted in other English regions, and if so, which elements comprise an effective implementation strategy.

Methods and analysis

Study Design

We will undertake a hybrid type 2 implementation-effectiveness study(29) to study the implementation of prison-hospital telemedicine in England. Hybrid type 2 studies have both a primary implementation outcome and a primary clinical outcome, and aim to generate evidence of a proven clinically effective intervention (prison telemedicine) on patient outcomes (access to secondary care) within a new context (English prisons).

The primary implementation outcome is the acceptability and feasibility of prison–hospital telemedicine in this context, and will be measured at different staff levels from different providers and for patients. The primary clinical effectiveness outcome is access to secondary care for people in prison in England, with secondary outcomes referring to the quality and cost of hospital care using this intervention.

Setting

Up to four prisons and three hospital settings in Surrey (England) will be included in the research, dependent on their telemedicine readiness during the study period. It is not possible to determine how many consultations will take place during the study period as hospital appointment numbers for prisoners are traditionally lower than the need within this population, given the barriers to accessing hospital care from prisons. The four prisons proposed include male and female prisoners, remand (not yet sentenced) and sentenced individuals, and different security categorisations.

Participants

Staff

There are two types of clinical provider staff who will be sampled in this project.

1. Prison healthcare staff who act as a contracted service within the Surrey prisons, but who are primarily employed by a central London NHS Foundation Trust. These staff will be responsible for referring patients to local community hospitals from prisons, local implementation and operation of the telemedicine system in prisons, attendance in telemedicine consultations with patients and liaison with prison staff for arrangement of patient transportation.
2. Hospital staff employed within Surrey community hospitals who will be delivering secondary care to patients in prisons over the telemedicine link. There are two Surrey hospitals who may be sampled for this research dependant on telemedicine readiness. Pre-implementation interviews will select staff from across all organisations. Throughout this protocol we refer to

For peer review only - http://bmjopen.bmj.com/site/about/guidelines.xhtml

these groups as: prison healthcare staff, hospital staff or all staff (prison and community hospital staff combined).

Wider prison staff from participating prisons will also take part as research participants. This will reflect their role in scheduling patient offsite transfers and escorting patients to the prison healthcare department for telemedicine appointment.

Staff sampling

Participants for interviews prior to and during implementation will be selected using a combination of purposive and snowball sampling to generate a key informant sample. Purposive sampling will identify initial interviewees known to have been involved in telemedicine implementation (e.g. Clinicians, Governance, IT). Snowball sampling will help identify other relevant interviewees unknown to the research team (e.g Commissioning leads, Strategic Directors) from those interviewed first. Interviewees will be selected from across all departments/prisons participating and will comprise a mix of staff at macro (board level), meso (departmental lead/managers) and micro levels (frontline delivery of telemedicine). This approach will result in a Key Informant sample,(30) guided both by the research teams' knowledge of staff involved in telemedicine implementation, and staff member's further knowledge on who is critical to support implementation locally. A maximum of 30 interviews is anticipated prior to telemedicine implementation, permitting a wide understanding of how telemedicine is understood and supported across varying staff levels and provider organisations. Mapping of staff at macro, meso and micro levels across hospital and prison healthcare organisations has informed this sample size. (31)

At the end of the telemedicine data collection period, semi-structured 1:1 interviews will be conducted with key staff informants from all clinical staff and wider prison staff (e.g. prison officers). Interviews will be split amongst a) staff who have experience of using/contributing to others use of telemedicine and b) staff from wider specialties who may wish to use telemedicine for prison consultations in the future. Purposive and snowball sampling will be used to identify staff interview participants with a strategic view of the health system who can comment on wider clinical specialties that may be appropriate for delivery via telemedicine in prisons, based on lessons learnt from implementation of the current services.

Prison staff (e.g. prison officers) from participating prisons will also be approached for participation in research activities, with participant numbers guided by the responsible prison Governor.

Prisoners

We will include prisoners from both telemedicine and telemedicine naïve prisons as participants within this study.

1. Patients in telemedicine prisons who use the system for an appointment will be invited by prison healthcare staff to complete a post appointment questionnaire and an opportunity to participate in a 1:1 interview with the research team.

For peer review only - http://bmjopen.bmj.com/site/about/guidelines.xhtml

2. In conjunction with the Prison Reform Trust charity (PRT) (32) we will organise and run focus groups (n=5) in five 'telemedicine naïve' prisons outside of the Surrey footprint (i.e. those who have no prior experience of telemedicine/ are not preparing for imminent telemedicine implementation). Participants will be recruited via existing PRT networks within the prisons.

Consent

Informed consent for participation from health care staff will be taken by the researcher. Informed consent from prisoners in focus groups will be taken by the Prison Reform Trust charity. Informed consent from prisoners in interviews will be taken by the researcher in the presence of a prison healthcare staff member.

Key Inclusion and Exclusion Criteria

Staff

For stage 1 staff interviews staff must have a stakeholder role in local telemedicine implementation and delivery. For stage 2 acceptability/feasibility interviews stuff must have either been involved in implementing telemedicine intervention in Surrey OR have used the intervention (either accompanying patients in consultations or providing secondary care treatment) OR work as a staff member in a prison offering telemedicine. For stage 2 interviews on wider telemedicine applicability staff must have either responsibility for prioritising or approving off-site appointments to hospitals for patients in prisons OR provide secondary care to offenders OR involved in healthcare delivery to prisons including strategic level staff.

Prisoners

Prisoner participation in any research activity in a prison is ultimately vetted and granted by prison staff in light of safety concerns regarding the individual. Several general exclusion criteria exist for participants in prisons which are common across all research activities proposed. These include: people who cannot give informed consent, people who are deemed a risk to researchers/not permitted to participate (by prison officials). In addition patients who move prison after their telemedicine consultation will not be approached for interview unless they have moved to another one of the study prisons where HMPPS ethical approval is valid.

To participate in patient questionnaires or patient interviews, the prisoner must have used the local telemedicine system. Participation in telemedicine focus groups will be open to all prisoners who are not excluded based on the criteria above and who currently reside in a telemedicine naïve prison.

Theoretical framework

Use of theory in implementation research both guides the collection and analysis of data and provides explanations for the phenomena observed.(33) Our implementation research will be informed primarily by Normalisation Process Theory (NPT)(34) which has been used to describe and explain implementation outcomes in numerous studies of complex healthcare interventions.(33)

For peer review only - http://bmjopen.bmj.com/site/about/guidelines.xhtml

For peer review only

Normalisation Process Theory focusses primarily on the work that individuals and groups undertake to operationalise and normalise an intervention. The theory proposes four constructs: Coherence, Cognitive Participation, Collective Action and Reflexive Monitoring.(34, 35) As identified in a systematic review of prison telemedicine,(12) staff attitude and behaviour is reported to be both the main driver and barrier to successful implementation, making NPT particularly relevant to this intervention. Successful implementation elsewhere has found that staff at macro (board level), meso (departmental lead/managers) and micro levels (frontline delivery of telemedicine) must be engaged and enthused in the provision of the intervention, across a multiplicity of providers (community hospital, prison healthcare, wider prison staff) all who have differing needs, wants and beliefs. (12)

Most proposed evaluation activities will focus on staff perceptions of feasibility and acceptability. Data collection (e.g. interview topic guides) will be guided by the constructs of NPT and research findings will be reported using a mixed methods approach under NPT construct headings.

Normalisation Process Theory calls for a description of the intervention and the context in which it will be deployed. Through interviews and use of the NOMAD survey(35) we will gather staff data on these factors. NPT has previously been criticised for its focus on individual and collective agency, and not paying enough attention to the wider organisational and relational contexts of the implementation.(33) Therefore, to provide more generalisable contextual information, in parallel to NPT, several constructs from the Consolidated Framework for Implementation Research (CFIR) will be used to guide an in-depth description of the intervention itself (core components and adaptable peripheries), a description of the outer setting (economic, political, and social context) and the inner setting (structural, political, and cultural contexts through which the implementation process will proceed).(36) In this way CFIR will be used to identify potential local contextual implementation barriers and facilitators that will be specifically explored within the individual/collective domains of NPT, which in itself will provide explanation as to why these matter in this context and the interrelations amongst constructs.

NPT refers to the opinions and reflections of staff involved in the intervention under study and the work they undertake to deliver the intervention. In the general community, patients are also required to undertake 'work' to access healthcare appointments. For example, booking the appointment, attending the appointment and remembering to take their medication. However, given the limited autonomy of patients in prison their ability to perform this 'work' is restricted. This means that NPT does not necessarily form the best framework for understanding prisoner data, despite the fact that their views on telemedicine will indicate whether it has a chance of normalisation (i.e. use). Prisoners will always be able to opt for standard care as usual instead of telemedicine. For this reason, prisoner data will be analysed using the Theoretical Framework of Acceptability (TFA) (37) which can be used to access prospective and retrospective acceptability of interventions to recipients where autonomy may be constrained. TFA constructs include: Affective Attitude, Burden, Ethicality, Intervention Coherence, Opportunity Costs, Perceived Effectiveness and Self-Efficacy. These will be used to guide both pre and post implementation data collection with patients.

For peer review only - http://bmjopen.bmj.com/site/about/guidelines.xhtml

Patient and Public Involvement

Two PPI representatives with lived experience of imprisonment assisted with preparation of study materials prior to ethical submissions and attend steering group meetings in the community. Following NRC approval PPI groups are being convened within prisons (1 male, 1 female) to input into ongoing research activities e.g. patient questionnaire design. These groups will each meet three times during the study, with continuity of membership if possible within the prison environment.

Research Activities and Quality Improvement measures

Research activities planned are shown in Figure 1. Several quality improvement measures will run in parallel to research activities and complement the final analysis.

Figure 1

Pre-implementation Research Activities (RAs)

RA1: Staff Interviews (Stage 1)

1:1 semi-structured interviews (maximum n=30) amongst telemedicine stakeholders from all staff groups to understand anticipated benefits, barriers and enablers to telemedicine usage in a prison-hospital setting, within the English commissioning context. The interview guide has been developed based on the NPT constructs (See Supplementary file 1)These constructs provide a framework with which to understand the sense-making of the intervention (telemedicine) amongst staff groups, the work they perceive will be involved in delivering the intervention and the benefits potentially accrued to system partners. Interview questions are aligned to those posed within the NOMAD questionnaire (see RA2), a survey measure of NPT, but allow participants to expand widely on their answers to provide rich qualitative understanding.

RA2: NOMAD questionnaire

The NOMAD questionnaire is an existing validated questionnaire, based on the principles of normalisation process theory (Finch et al., 2018). The NOMAD questionnaire seeks to identify barriers to normalisation of an intervention in healthcare to explain why interventions fail to embed in day to day use. We will administer this anonymous email survey questionnaire to all healthcare staff and additionally to prison officers at participating prisons. This will collect data on staff perceptions and concerns surrounding telemedicine. This questionnaire will be administered prior to telemedicine implementation and several months after implementation, and the changes in response quantified.

For peer review only - http://bmjopen.bmj.com/site/about/guidelines.xhtml

RA5: Prison Focus Groups

Study prisons have been advocating for telemedicine introduction for several years and prisoners are aware it may soon be introduced. We will collect data to represent broader prisoner views on telemedicine in prison focus groups (n=5) where there is currently no 'threat' of implementation and prisoners will be free to speak openly of their concerns. Results will be used where applicable to inform the current telemedicine service implementation and to inform policy for future telemedicine services if research results suggest it can be widely implemented. For example: "What do you think would stop people wanting to use a telemedicine system and how could we mitigate this?" The topic guide for focus groups has been guided by the TFA and PPI groups (see Supplementary File 2) The first topic guide iteration was prepared by the researcher, by applying TFA constructs (Affective Attitude, Burden, Ethicality, Intervention Coherence, Opportunity Costs, Perceived Effectiveness and Self-Efficacy) to the perceived telemedicine model in prison. For example, an opportunity cost to attending a telemedicine appointment may be missing a family visit or the opportunity to travel outside the prison walls. This topic guide was reviewed at two PPI groups (one male, one female) and refined in line with feedback.

Implementation Research Activities

These activities will commence after telemedicine has become operational in at least one prison site.

RA1: Staff Interviews (Stage 1) continued

Frontline delivery staff will be re-interviewed during implementation to understand how the telemedicine services proposed at outset have changed and adapted as issues arise, and what specific barriers have arisen that were not anticipated. Staff will be approached for re-interview when the system has been running for a minimum of four months. They will be re-interviewed once only as part of RA1, however may also be interviewed during RA7 (staff interviews at end of data collection period). Only staff involved in frontline service delivery will be re-interviewed for RA1, and we anticipate a maximum of 10 interviews based on knowledge of staff numbers involved in initial telemedicine delivery.

RA3: Clinician questionnaire on telemedicine appointment

Both hospital clinicians and prison healthcare staff involved in a telemedicine appointment will complete a paper questionnaire at the close of each appointment. This will seek to understand whether the appointment was clinically acceptable, with reference to anticipated barriers identified in the staff interviews at outset. The content of the questionnaire will include questions from the Telemedicine Usability Questionnaire (TUQ)(38) which relate to staff actions for use of telemedicine. For example, dialling in to the telemedicine system will be the responsibility of the prison healthcare staff member and not the patient. Questions will also be informed by the literature review that precedes this protocol on enablers/barriers to prison telemedicine,(12) for example "where you able to access prison electronic health records contemporaneously?".

For peer review only - http://bmjopen.bmj.com/site/about/guidelines.xhtml

The questionnaire will be refined after clinical staff interviews have been conducted, to include questions around staff concerns identified. We currently anticipate the questions shown in Figure 2.

Figure 2

RA4: Patient questionnaire on telemedicine appointment

All patients who have a telemedicine appointment will be offered a short, anonymous paper questionnaire immediately after their appointment finishes, to collect data on patient acceptability of telemedicine appointments. Questionnaires should provide information that may be overlooked as 'not important' or not recalled in later patient interviews e.g. *"Did the doctor speak over you?"*

The content of the questionnaire will be based on the Telemedicine Usability Questionnaire (TUQ)(38) with several additional questions pertaining to appointments in prison. For example, *"Were you able to get to your appointment on time?"* which refers to the fact that prison officers will need to escort patients to their telemedicine appointment within the prison healthcare department.

The questionnaire will be finalised with input from PPI groups. For example, recent PPI work has suggested questions including, "*Have you used a video court link before?"* and *"Did the healthcare staff member dial in before you were in the room?"* are likely to be particularly relevant to patient experience of prison telemedicine

RA6: Patient 1:1 interviews

1:1 semi-structured interviews (n=15) will be held with patients who have had a telemedicine consultation to collect information on patient experience and acceptability. Where applicable participants will be asked to compare telemedicine treatment to previous offsite secondary care appointments. It is not possible to determine in advance how many patients will have previous offsite secondary care experience given the dynamic nature of the prison population.

Patients who are offered the opportunity for a telemedicine appointment but do not choose to undertake it will be approached for interview to understand their views of telemedicine.

RA7: Staff interviews (stage 2)

Interviews with staff who have used the telemedicine system with seek to understand the feasibility and acceptability of the current delivery model. Interview topic guides will be informed by NPT constructs and results of the NOMAD survey previously administered.

All frontline healthcare staff who have used the model will be invited to participate in interview, as well as staff involved in operational or managerial support of the model (n=10-15). It is possible that prison healthcare teams may choose to assign only one or two individuals to chaperone telemedicine consultations during the mobilisation phase to ensure consistency, which may limit the interview sample size.

For peer review only - http://bmjopen.bmj.com/site/about/guidelines.xhtml

Qualitative analysis

All qualitative data collection activities will be recorded on an encrypted digital voice recorder and professionally transcribed.

Focus group and interview data will be analysed using Framework Analysis, by which data is sifted, charted and sorted in accordance with key issues and themes. Framework analysis involves a five step process: 1. familiarisation; 2. identifying a thematic framework; 3. indexing; 4. charting; and 5. mapping and interpretation. (39)

Quality Improvement (QI) Measures

The NHS Friends and Family Test

Alongside the research activities a standard NHS QI tool (the Friends and Family test) will be administered by the prison healthcare staff to patients, to monitor satisfaction of telemedicine services for all appointments. Anonymised data from this will be provided to the research team to supplement the research summary and will be compared to pre-telemedicine satisfaction scores.

Quantitative Service Evaluation - Pre and Post Anonymised Health Records Data for Specialties under Study

After nine months of service delivery routine service evaluation data will be used to assess clinical effectiveness, specifically to understand whether appointments happened more quickly using telemedicine and whether cancellations were reduced. This data will be collected and analysed by the NHS as part of their routine clinical evaluation of a service change. Aggregate anonymised electronic health records data will be provided to the research team to supplement the research summary including: number of secondary care consultations and referrals, time from referral to treatment, total number of off-site transfers, number of and reason/s for appointment cancellation. This data will be compared at stages throughout implementation to pre-telemedicine data on the same indicators, to account for a transition phase as the service becomes established.

Quantitative analysis

We will seek to understand how the use of telemedicine changes the referral to treatment time for patients and the number of appointment cancellations. We will also compare this to equivalent indicators for patients from the general community to understand whether telemedicine can improve equity between these groups.

We will collect information currently lacking such as the distributional form and standard deviation of key indicators of healthcare use such as referral to treatment time. This information can then be used in future research to calculate sample size and power as well as determining the most appropriate statistical tests to use in analysing the data. From the basic information on the number of cases we

For peer review only - http://bmjopen.bmj.com/site/about/guidelines.xhtml

might reasonably expect to use telemedicine for gastroenterology within one prison over the period of the study (n=50-55), we can state that if the intervention (telemedicine) has a medium to large effect size, the feasibility study will be powered to detect this difference, even with the relatively small sample size. Specifically, we estimate a value of 0.6 for Cohen's D which he equates to medium to large effect size.(40) This data will provide evidence as to whether telemedicine can provide a difference in care.

Service Evaluation to Inform Wider Specialties for Telemedicine Delivery

To understand which other specialties may be a priority for prison telemedicine delivery aggregate, anonymised prison healthcare records data will be provided to the research team. This will provide information on the need and distribution of all secondary care outpatient appointments for people in participating prisons including: hospital and specialty referred to e.g. cardiology, time between referral and appointment by specialty, cost of prison officer escorts for appointment and cancellation data (if applicable), i.e. reason for cancellation and date of cancellation by specialty.

Indicators will be compared as proportions to allow for differences in prison population sizes and the dynamic population cohort. For example, number of cancelled appointments or number of referrals made to a hospital specialty will be presented per 100 standard GP appointments within the prison.

This analysis will help inform which other hospital specialties may be areas of value for telemedicine development e.g. department with most referrals, department with longest wait, department with most cancellations. This will complement stage 2 staff interviews on wider telemedicine specialties.

Use of data collection tools throughout this research is detailed further in table 1.

For peer review only

Table 1 Data collection instruments

DATA COLLECTION INSTRUMENT	ADMINISTERED TO	PURPOSE	POINT OF ADMINISTRATION	ADMINISTERED BY	ANALYSIS PLAN
**NOMAD (NPT) survey**	Staff involved in operation, delivery or support of prison telemedicine (hospital staff, prison healthcare staff, prison officers)	To understand staff pre-conceptions and concerns that may affect implementation and normalisation of telemedicine. This wider survey will complement in-depth staff interviews.	Prior to telemedicine implementation and repeated at end of telemedicine data collection period.	Online survey sent through email link. Link circulated to staff by provider/prison leads (e.g. Local Principle Investigators).	Descriptive analysis and comparison of survey data prior to/post implementation.<br><br>Descriptive analysis will be triangulated with qualitative data on NPT constructs (from staff interviews) to understand how telemedicine is perceived and understood by staff.
**Staff telemedicine questionnaire (Informed by the Telemedicine Usability Questionnaire)**	Staff either delivering or chaperoning prison telemedicine consultations (hospital staff, prison healthcare staff)	To understand the clinical acceptability and feasibility of telemedicine appointments to staff, limitations and staff time associated costs of telemedicine. This questionnaire will complement in-depth staff interviews.	To be completed by the staff member at the end of each telemedicine consultation	Paper questionnaire for self-completion available at study sites	Descriptive analysis of survey measures

For peer review only - http://bmjopen.bmj.com/site/about/guidelines.xhtml

**Patient telemedicine questionnaire (Informed by the Telemedicine Usability Questionnaire)**	Patients who have had a telemedicine appointment	To understand the acceptability of telemedicine appointments to patients. This questionnaire will complement in-depth patient interviews.	To be completed by the patient after a telemedicine consultation (can be taken away for completion)	Paper questionnaire for self-completion available at study sites. Prison staff or peer healthcare reps will assist with completion if required for literacy reasons.	Descriptive analysis of survey measures and qualitative analysis of free text answers in NVIVO software. Qualitative data from surveys will be analysed alongside interview data pertaining to patient experience following the principles of framework analysis.
**NHS Friends and Family Test**	All patients at the prison complete this tool as part of usual care. Survey data will be collated for prison telemedicine appointment subsets.	To understand whether patient satisfaction of healthcare improves with telemedicine	To be completed by the patient after a telemedicine consultation	Paper questionnaire for self-completion available at study sites.	Descriptive analysis and comparison to overall historic departmental trends in healthcare satisfaction.

For peer review only - http://bmjopen.bmj.com/site/about/guidelines.xhtml

For peer review only

Economic analysis

Cost modelling will be undertaken by a health economist to understand how the wider rollout of prison telemedicine across specialties may affect cost effectiveness of secondary care delivery to prisoners (including costs to prison healthcare provider, wider prison costs and NHS costs). Data on resource associated with delivery of telemedicine (e.g. staff time and processes) will be collected in staff feasibility/acceptability stage 2 interviews and on the staff questionnaires. For example, questionnaires will collect data on "*How long did you take to log on to the telemedicine system?"* and *"How long was the appointment you chaperoned?".* Interviews will collect data such as *"which staff grades are you comfortable with designating as a patient chaperone"* or *"what tasks did you forgo to chaperone a telemedicine appointment?".*

Individual prisons, the prison healthcare provider and community hospitals will provide costings data associated with the delivery of the telemedicine model compared with standard care which will be attributed to reported data.

Confidentiality

Qualitative data will be anonymised in published reports and upon archiving at the close of the study to protect participant confidentiality. Interview/focus group data will be recorded on an encrypted dictaphone. Service evaluation data will be provided as aggregate, anonymised data to remove the risk of deductive disclosure. Questionnaires from both staff and patients will be anonymous.

Ethics and Dissemination

This study has received ethical approval from the London South East NHS Research Ethics Committee (19/LO/0098), the HMPPS National Research Committee (NRC 2018-212) and Health Research Authority and Health and Care Research Wales (HRA IRAS 229646).

Study results will be published in peer reviewed journals and presented at academic conferences. Wider dissemination will be achieved through health and justice networks/organisations such as Public Health England, The Prison Reform Trust and the Royal College of General Practitioners Secure Environment Group.

Ongoing feedback will be provided to prison Governors by the lead researcher, both verbally and by email communication. The Prison Reform Trust will ensure research summaries are made available that are accessible to people in prison.

Discussion

The potential for the prison context to restrict research activities should not be underestimated. Undertaking research in prisons is complex given the restrictive nature of the environment and the

For peer review only - http://bmjopen.bmj.com/site/about/guidelines.xhtml

For peer review only

need to operate within the prison regime, which will inevitably differ by establishment. Health research approvals must be granted by the research sponsor, an NHS research ethics committee, the Health Research Authority (who provide general health service agreement) and individual NHS hospitals. In addition, for research taking place in prisons the HMPPS National Research Committee must provide approval which, alongside methodological scrutiny, they grant in line with their guidance:

"All research must be of benefit to HMPPS and the links to HMPPS priorities must be explicit."(41)

Finally individual Prison Governors must provide agreement that research can take place within their given establishment.

Data collection is also more challenging than in community settings. Prison electronic health records currently sit separately from community NHS records and are jointly controlled by both prison healthcare provider and NHS prison commissioners. Data collection direct from prisoners is reliant on the ability to access patients. When using questionnaires these must account for a population which is likely to have low literacy levels and an element of mistrust in research. In addition, as identified in our forthcoming literature review,(12) prison and hospital healthcare providers are likely to anticipate and experience different and unequal benefits, barriers and facilitators which may affect enthusiasm for implementation.

To undertake this research, the Principal Investigator (PI) is assuming the role of an embedded researcher, which is known to present challenges to rigorous research within healthcare organisations.(42) In this instance the PI will be embedded both within the community healthcare team/s and the prison healthcare team alongside their academic base. This may compromise their ability to truly embrace 'dual' affiliation and relationship building given that relationships must be formed with two different hospital provider teams.

In summary, a telemedicine model straddling community and prison healthcare providers, situated within the context of the English prison system represents a highly complex intervention. For this reason, we have determined it appropriate to only study prisons within one county of England, which houses both male and female prisons of different security categorisation. This may restrict our sample size; however, attempts to widen the study to other counties would introduce other prisons, other hospitals, other providers and other commissioners and leave the study prone to failure as we strive to understand whether successful implementation is possible.

Potential Significance of Research

If established effectively, prison-hospital telemedicine offers the potential to improve access and quality of secondary care for prisoners, and to reduce NHS costs by reducing the amount of offsite escorts from prison to hospital that must be reimbursed from public NHS funds. There is an opportunity cost to all NHS spending, with one funded service or treatment representing an opportunity foregone to invest elsewhere.

For peer review only - http://bmjopen.bmj.com/site/about/guidelines.xhtml

Prisoners experience a disproportionately higher burden of disease than people within the general community including infectious diseases, long-term conditions and mental health problems.(43) The prison population is typically characterised as younger but the number of older prisoners continues to rise, bringing new challenges in the form of treatment of multiple co-morbidities within prison environments.(44) Telemedicine may be one approach to improve the health of people in prisons, which can in turn bring community dividends such as reducing reoffending and a reduction in the wider reservoir of infectious disease.(45, 46) However, it cannot hope to bring these benefits for a traditionally under-served and under-researched population, unless the implementation is successful.

In addition, the study of implementation science within criminal justice settings is highly limited. At the time of writing this protocol, we have identified only three articles pertaining to implementation research in health and justice settings.(47-49) This study will therefore contribute knowledge to an emergent and complex field of study.

Declarations

Ethics approvals

This study has received ethical approval from the London South East NHS Research Ethics Committee (19/LO/0098), the HMPPS National Research Committee (NRC 2018-212) and Health Research Authority and Health and Care Research Wales (HRA IRAS 229646).

Study Sponsor

University College London (Joint Research office) email: uclh.jro-communications@nhs.net

Competing interests

The authors declare no competing interests.

Funding

CE is funded by a National Institute for Health Research (NIHR), (Clinical Doctoral Research Fellowship ICA-CDRF-2017-03-006) for this research project. JG is partially funded by a Health Education England/National Institute of Health Research Clinical Lectureship (ICA-CL-2016-02-024). AH is an NIHR senior investigator. This publication presents independent research funded by the National Institute for Health Research (NIHR). The views expressed are those of the author(s) and not necessarily those of the NHS, the NIHR or the Department of Health and Social Care. The funder and sponsor has no role in study data collection, management, analysis, interpretation or writing/decisions to submit publications.

Authors' contributions

CE conceived the project. CE wrote the original research grant funding application and protocol, with methodological guidance and support from academic supervisors AH, JG and GB. SE, SP, MG, AA are senior clinicians within participating hospitals who have guided methodological considerations throughout and provide clinical supervision of the PhD student. SP and MG are named local Principle Investigators, AH is the named Chief Investigator for the study for ethical approvals. CE is the named

For peer review only - http://bmjopen.bmj.com/site/about/guidelines.xhtml

Principle Investigator and PhD student. All authors reviewed and commented on the final draft of this protocol.

Acknowledgements

We are grateful to the University College London Joint Research Office, and the Research and Development departments at Royal Surrey County Hospital NHS Foundation Trust and Central North West London NHS Foundation Trust, for their support and assistance in developing this research study.

Authors' information (optional)

CE is undertaking this study as a PhD study which forms the basis of her NIHR Clinical Doctoral Research Fellowship. The named Chief Investigator for the study is her primary PhD supervisor AH in line with University College London study sponsorship policy.

Bibliography

1. Charles A, Draper H. 'Equivalence of care' in prison medicine: is equivalence of process the right measure of equity? J Med Ethics. 2012;38(4):215.
2. Assembly UG. Nelson Mandela Rules. United Nations; 2015.
3. Leaman J. Rapid review of evidence of the impact on health outcomes of NHS commissioned health services for people in secure and detained settings to inform future health interventions and prioritisation in England. In: England PH, editor. London, UK2016.
4. Association BM. The medical role in restraint and control: custodial settings. London, UK: British Medical Association; 2009.
5. Bara C, Maillard H, Beneton N, Serre P, Celerier P. Teledermatology in the management of skin diseases in prison inmates: Experience in central France. [French]. Ann Dermatol Venereol. 2013;140(3):216-8.
6. Blanchet K. Innovative programs in telemedicine: University of Texas Medical Branch at Galveston Electronic Health Network. Telemed J e-Health. 2005;11(2):116-23.
7. Braniff C, Connelly C, Mollison L. Telemedicine allows successful Hepatitis C treatment in Western Australian prisons. J Viral Hepatitis. 2015;22:90-.
8. Chipps J, Ramlall S, Mars M. Practice guidelines for videoconference-based telepsychiatry in South Africa. Afr J Psychiatry. 2012;15(4):271-82.
9. Fallon PV. Introducing telehealth to English prisons. Int J Prison Health. 2007;3(1):77-8.
10. Gualano MR, Bert F, Andriolo V, Grosso M, Minniti D, Siliquini R. Use of telemedicine in the European penitentiaries: current scenario and best practices. Eur J Public Health. 2017;27(1):30-5.
11. Lee YM. A debate about telemedicine in South Korea. J Korean Med Assoc. 2016;59(11):828-31.
12. Edge C, Black, G., King, E., George, J., Patel, S., Hayward, A. . Improving care quality with prison telemedicine: the effects of context and multiplicity on successful implementation and use. The Journal of Telemedicine and Telecare. 2019;1357633X19869131. doi: 10.1177/1357633X19869131.
13. Wheeler J, Hinton E. Effectiveness of telehealth on correctional facility health care: A systematic review protocol. JBI Database Syst. 2017;15(5):1256-64.
14. Caudill RL, Sager Z. Institutionally based videoconferencing. Int Rev Psychiatry. 2015;27(6):496-503.

For peer review only - http://bmjopen.bmj.com/site/about/guidelines.xhtml

15. Garfield MJ, Watson RT. Four case studies in state-supported telemedicine initiatives. Telemed J e-Health. 2003;9(2):197-205.
16. Lyckhoml LJ, Justis D. Delivery of cancer care to inmates of correctional facilities through telemedicine. Journal of Clinical Oncology; Jul2004. p. 764S-S.
17. Magaletta PR, Fagan TJ, Peyrot MF. Telehealth in the federal bureau of prisons: Inmates' perceptions. Prof Psychol-Res Pract. 2000;31(5):497-502.
18. Salem Y, editor Forensic telepsychiatry in UK. European Psychiatry Conference: 18th European Congress of Psychiatry Munich Germany Conference Publication:; 2010.
19. Pearce L. Nurses lead the way with webcam consultations. Nurs Stand. 2017;32(2):22-3.
20. Clements S. On the challenge of providing care for prisoners. HSJ. 2012;122(6331):Suppl 10.
21. NHS. The NHS Long Term Plan. UK; 2019.
22. Leonard S. The development and evaluation of a telepsychiatry service for prisoners. J Psychiatr Ment Health Nurs. 2004;11(4):461-8.
23. Service NOM. National Security Framework Ref: NSF 6.6 SECURITY MANAGEMENT FUNCTION Management and Security of Escape List (E-List) Prisoners. 2015.
24. Stewart R. Urgent Notice - HMP Birmingham. In: Justice Mo, editor. London2018.
25. Committee PHaSC. 12th report - Prison Health. www.parliament.uk2018.
26. ONS. Population Estimates. UK: Office of National Statistics; 2017.
27. Surrey-i. Surrey Prison Population. Surrey, UK: Surrey County Council; 2019.
28. England N. Integrated Care Systems [Available from: https://www.england.nhs.uk/integratedcare/integrated-care-systems/.
29. Curran GM, Bauer M, Mittman B, Pyne JM, Stetler C. Effectiveness-implementation hybrid designs: combining elements of clinical effectiveness and implementation research to enhance public health impact. Med Care. 2012;50(3):217-26.
30. Marshall MN. The key informant technique. Family Practice. 1996;13(1):92-7.
31. Sandelowski M. Sample size in qualitative research. Res Nurs Health. 1995;18(2):179-83.
32. Trust PR. Prison Reform Trust Website UK2019 [Available from: http://www.prisonreformtrust.org.uk/.
33. May CR, Cummings A, Girling M, Bracher M, Mair FS, May CM, et al. Using Normalization Process Theory in feasibility studies and process evaluations of complex healthcare interventions: a systematic review. Implement Sci. 2018;13(1):80.
34. Murray E, Treweek S, Pope C, MacFarlane A, Ballini L, Dowrick C, et al. Normalisation process theory: a framework for developing, evaluating and implementing complex interventions. BMC Medicine. 2010;8(1):63.
35. Finch TL, Girling, M., May, C.R., Mair, F.S., Murray, E., Treweek, S., Steen, I.N., McColl, E.M., Dickinson, C., Rapley, T. NoMad: Implementation measure based on Normalization Process Theory. [Measurement instrument]. 2015.
36. Damschroder LJ, Aron DC, Keith RE, Kirsh SR, Alexander JA, Lowery JC. Fostering implementation of health services research findings into practice: a consolidated framework for advancing implementation science. Implement Sci. 2009;4(1):50.
37. Sekhon M, Cartwright M, Francis JJ. Acceptability of healthcare interventions: an overview of reviews and development of a theoretical framework. BMC Health Serv Res. 2017;17(1):88.
38. Parmanto B, Lewis AN, Jr., Graham KM, Bertolet MH. Development of the Telehealth Usability Questionnaire (TUQ). International journal of telerehabilitation. 2016;8(1):3-10.
39. Ritchie JS, L. Qualitative data analysis for applied policy research: Taylor & Francis books; 1994.
40. Cohen J. Statistical Power Analysis for the Behavioral Sciences, Second Edition. United States of America: Lawrence Erlbaum Associates; 1988.
41. HMPPS. National Research Committee – Application Guidance. In: Service HMsPaP, editor. London, England.
42. Vindrola-Padros C, Pape T, Utley M, Fulop NJ. The role of embedded research in quality improvement: a narrative review. BMJ Quality & Safety. 2017;26(1):70.

For peer review only - http://bmjopen.bmj.com/site/about/guidelines.xhtml

43. Aldridge RW, Story A, Hwang SW, Nordentoft M, Luchenski SA, Hartwell G, et al. Morbidity and mortality in homeless individuals, prisoners, sex workers, and individuals with substance use disorders in high-income countries: a systematic review and meta-analysis. Lancet. 2018;391(10117):241-50.
44. Sturge G. UK Prison Population Statistics: Briefing Paper House of Commons Library; 2018.
45. RCGP-SEG. Equivalence of care in Secure Environments in the UK - Position Statement. Royal College of General Practitioners Secure Environments Group; 2018.
46. O'Moore E. England PH, editor. Gov.UK2015. Available from: https://publichealthmatters.blog.gov.uk/2015/07/06/the-community-dividend-why-improving-prisoner-health-is-essential-for-public-health/.
47. Pearson M, Brand SL, Quinn C, Shaw J, Maguire M, Michie S, et al. Using realist review to inform intervention development: methodological illustration and conceptual platform for collaborative care in offender mental health. Implement Sci. 2015;10(1):134.
48. Almost J, Gifford WA, Doran D, Ogilvie L, Miller C, Rose DN, et al. Correctional nursing: a study protocol to develop an educational intervention to optimize nursing practice in a unique context. Implement Sci. 2013;8(1):71.
49. Johnson JE, Wiltsey-Stirman S, Stout R, Miller T, Zlotnick C. Implementation of evidence-based mental health practices in criminal justice settings: Implications from a Hybrid Type I cost-effectiveness trial. Implement Sci. 2015;10(1):A76.

For peer review only

For peer review only - http://bmjopen.bmj.com/site/about/guidelines.xhtml

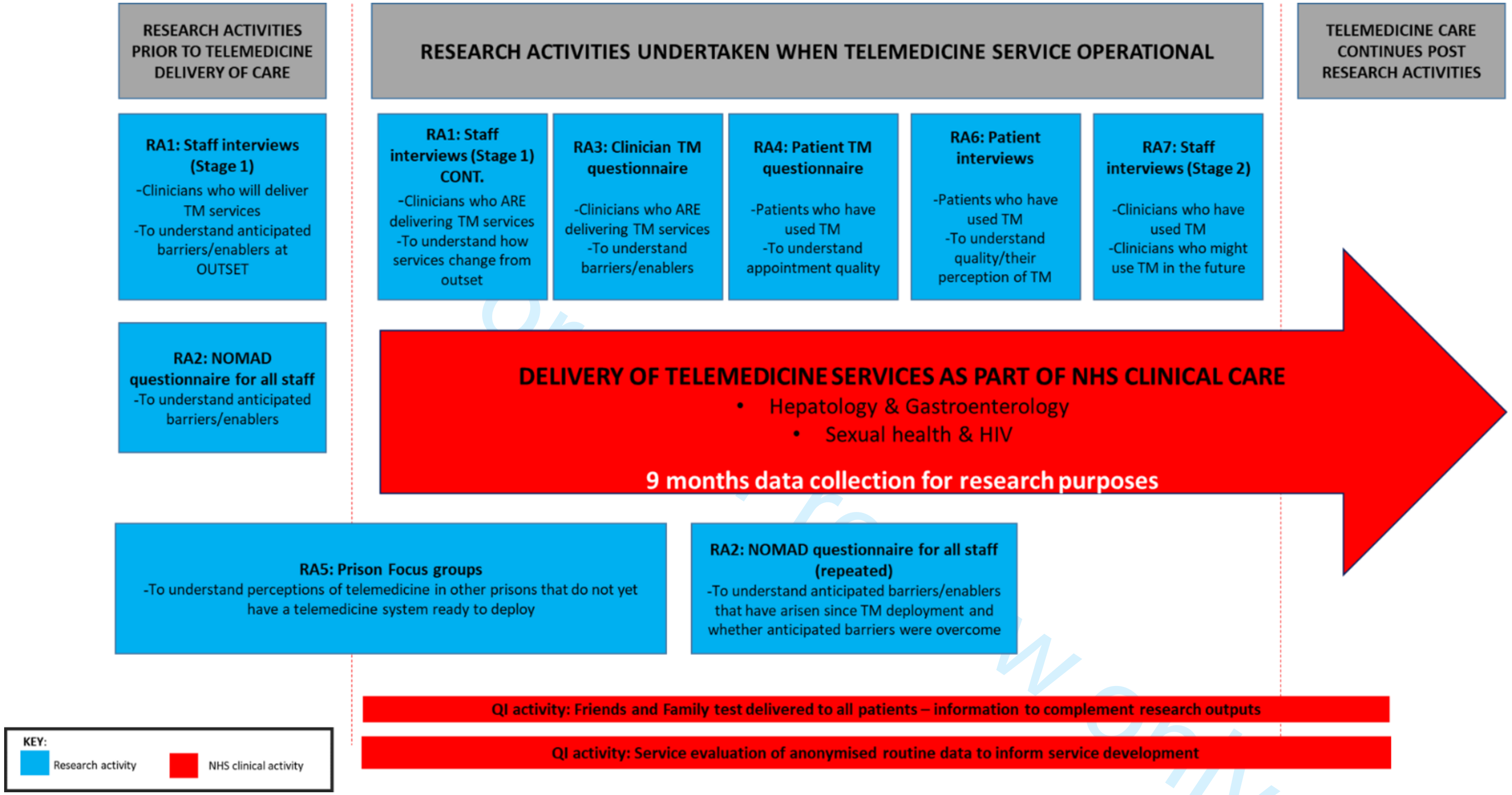

Figure 1 Study Research Activities

- Were you able to connect/log on to the system with ease?
- Was the quality of the internet connection sufficient for consultation?
- Was the telemedicine consultation sufficient to understand/treat patient clinical issues (or will an in-person appointment be required)?
- Were you able to discuss the onward treatment plan for the patient with the hospital/prison staff member?
- Would there have been difficulties in planning/delivering the patient treatment plan if you had not spoken directly with the hospital/prison clinical staff?
- Did the patient need your help to clarify any clinical information during or after the appointment?
- Did the patient appear comfortable in the telemedicine appointment?
- In hindsight, do you think the patient was appropriately referred to a telemedicine appointment? (and if not, why not?)

Figure 2 Anticipated staff questions for questionnaire post telemedicine consultation

For peer review only

STAFF TOPIC GUIDE PRISON TELEMEDICINE – Prior to telemedicine implementation

Introduction, consent taken

Organisational/ inter-relationships

- How does prison health/secondary care access fit into your organisational priorities/culture/norms?
- How does telemedicine fit into your organisational priorities/culture/norms?
- Thinking about national or local policies now, how do they influence your decision to introduce telemedicine?
- How networked would you say your organisation is with (relevant prisons/hospital)?

General background to telemedicine in prisons

We are planning to introduce telemedicine in HMP xxxx.

- What are your views on/what do you think about telemedicine?
- Why do you think we are planning on introducing telemedicine?
    - *FOR HOSPITALS ONLY – do you know of any barriers to accessing secondary care for prisoners which might influence the decision to introduce telemedicine ?*

Telemedicine in practice – working perspective

- How do you perceive your role in progressing telemedicine between hospitals and prisons?
    - *How does this fit with your current role?*
- Talk me through how you think a telemedicine consultation might work in practice
    - *What are the pros and cons of this consultation?*
    - *What else has to happen to make sure this consultation takes place successfully?*
- How might telemedicine impact on the work of other people in your organisation?
- How might your organisation react to this type of innovation? – why?
    - *What about the wider political/ professional system?*
- What has been your experience of introducing telemedicine so far?
- If we were to launch telemedicine tomorrow, is there anything else you think would get in the way?

Version 1 Date: 20/12/2019
Staff interview topic guide – prior to telemedicine implementation

For peer review only

Telemedicine benefits

- How might telemedicine affect you and your role?
- How might telemedicine affect patients in prisons?
- How might telemedicine affect your wider organisation (as opposed to just you or the patient)?
- How might telemedicine affect other associated partners (e.g. HMPPS/NHSE)?
- How might telemedicine affect you personally? (eg Extrinsic incentives such as goal-sharing awards, performance reviews, promotions, and raises in salary, and less tangible incentives such as increased stature or respect).

Telemedicine in practice – technological perspective

- What would an ideal system look like technology-wise?
- How easy or hard is it to work with other departments in your organisation to get the technology set up and ready to use?
- What might make people reluctant to use the telemedicine system (technology wise)?

Wrap up

Is there anything else you would like to add about prison-hospital telemedicine?

Thank and close

Version 1 Date: 20/12/2019
Staff interview topic guide – prior to telemedicine implementation

For peer review only

Using Video Consultations for Hospital Appointments in Prison – Focus Group Topic Guide

Introduction:

We are running a research project to see if using video consultations for hospital appointments in prisons makes healthcare better.

It can take a long time to get seen by a doctor at the hospital if you are in prison because only a few patients can go off-site every day. If we can do some appointments over the internet using a webcam patients might get seen quicker. It would also leave the hospital transfers for people who can't be seen over video consultation. We want to test whether prisons can do hospital appointments using video consultation. We want to know if this makes healthcare better for patients, and whether it helps the NHS too.

There are no plans we know of to try and use video consultations in HMP xxx at the moment, but we would like to gather your thoughts and feedback on this idea. We want to know what you would be worried about and how you think it should be used to make it a good experience.

For peer review only

For peer review only - http://bmjopen.bmj.com/site/about/guidelines.xhtml

Timing	Section	Notes
	**Focus Group Opener**	
10 mins	I'd like to start with a bit of discussion around video consultations in prison.<br><br>**What were your initial thoughts when you first heard about the topic of this group?**<br><br>**How would you feel if you were offered a video consultation tomorrow?**<br><br>Essentially video appointments will be similar to the video court link process. You'll get a choice of whether to use the video system at the prison or wait for a face to face appointment.<br><br>**What is the best way we could name/describe these appointments?**	*after discussion provide further detail around what these consultations will hypothetically look like (e.g healthcare staff member in the room) and how appointments will be booked etc*
	**Concerns and benefits**	
30 mins	**What are your initial thoughts on video appointments for hospital care?**<br><br>• **What do you think people will see as benefits of video appointments?**<br><br>• **What do you think people will be concerned about in regards to video appointments?**<br><br>• **(if not already covered) Why do you think some people might refuse to have one?**	*After discussion if not already brought up, explain other health benefits e.g. shorter appointment waits, no prison officer in consultation, more dignity*
	**When would/wouldn't you choose it?**	
20 mins	We understand that even if people are generally OK with the idea of video appointments, there will be some circumstances where they still want to choose a face to face appointment.<br><br>**When might people choose to have a video appointment?**	*e.g. feeling really bad and want a quicker appointment, been waiting ages already, less*

IRAS study number: 229646, Version 1.1, 03/10/19

For peer review only - http://bmjopen.bmj.com/site/about/guidelines.xhtml

	When might people choose not to have a video appointment?	*worrisome health condition, private condition that you don't want PO to hear about*<br><br>*e.g. certain health conditions*
	**Opting in to telemedicine appointments**	
20 mins	People will always have the choice of having a normal face to face appointment if they get offered a video appointment. We want people to make a truly informed choice about which is best for them.<br><br>**Based on what we have just discussed, what do people need to know about video appointments to make an informed choice to have one or not?**<br><br>• **Do you think people will have confidence in the system?**<br><br>• **What would give people confidence in the system?**<br><br>**If people decide to opt for a video appointment, what information do they need before they have the actual appointment?**<br><br>**How could we best promote video appointments?**<br><br>**How should we provide 'mythbusting' information about them?**	*e.g. privacy, computer will work, Dr can diagnose ok over internet?*<br><br>*e.g. peer champions, leaflets*<br><br>*e.g. what time your appointment is, how to get there, what happens if the computer isn't working – how long is the wait until the next appointment etc?*<br><br>*e.g. leaflet, prison radio, peer advisors*
	**Having an appointment**	
15 mins	Unlike with outside appointments, you should know the time and date of your appointment in advance. Hopefully this will allow you to prepare any questions or worries you want to ask the hospital doctor. So let's imagine it's the day of your appointment<br><br>**Can you imagine what it will be like to participate in your appointment?**	*e.g. hard to get to healthcare, hard to prepare questions, hard to*

For peer review only - http://bmjopen.bmj.com/site/about/guidelines.xhtml

	**How do you think you would feel during your video appointment?**	*engage with doctor over internet*<br><br>*e.g. confidence, that you can engage well and explain yourself over video link, that you would be understood*
	**Wrap up and close**	
15 mins	**Is there anything else you want to add about video appointments in prisons?**<br><br>Thanks – further follow up details etc.	

For peer review only

For peer review only - http://bmjopen.bmj.com/site/about/guidelines.xhtml

Standards for Reporting Qualitative Research (SRQR)*

http://www.equator-network.org/reporting-guidelines/srqr/

Title and abstract

	Page/line no(s).
**Title** - Concise description of the nature and topic of the study Identifying the study as qualitative or indicating the approach (e.g., ethnography, grounded theory) or data collection methods (e.g., interview, focus group) is recommended	1
**Abstract** - Summary of key elements of the study using the abstract format of the intended publication; typically includes background, purpose, methods, results, and conclusions	1-2

Introduction

**Problem formulation** - Description and significance of the problem/phenomenon studied; review of relevant theory and empirical work; problem statement	3-4
**Purpose or research question** - Purpose of the study and specific objectives or questions	4

Methods

**Qualitative approach and research paradigm** - Qualitative approach (e.g., ethnography, grounded theory, case study, phenomenology, narrative research) and guiding theory if appropriate; identifying the research paradigm (e.g., postpositivist, constructivist/ interpretivist) is also recommended; rationale**	4, 7
**Researcher characteristics and reflexivity** - Researchers' characteristics that may influence the research, including personal attributes, qualifications/experience, relationship with participants, assumptions, and/or presuppositions; potential or actual interaction between researchers' characteristics and the research questions, approach, methods, results, and/or transferability	14
**Context** - Setting/site and salient contextual factors; rationale**	5
**Sampling strategy** - How and why research participants, documents, or events were selected; criteria for deciding when no further sampling was necessary (e.g., sampling saturation); rationale**	5-6
**Ethical issues pertaining to human subjects** - Documentation of approval by an appropriate ethics review board and participant consent, or explanation for lack thereof; other confidentiality and data security issues	6, 13
**Data collection methods** - Types of data collected; details of data collection procedures including (as appropriate) start and stop dates of data collection and analysis, iterative process, triangulation of sources/methods, and modification of procedures in response to evolving study findings; rationale**	8-13

For peer review only - http://bmjopen.bmj.com/site/about/guidelines.xhtml

**Data collection instruments and technologies** - Description of instruments (e.g., interview guides, questionnaires) and devices (e.g., audio recorders) used for data collection; if/how the instrument(s) changed over the course of the study	Table 1
**Units of study** - Number and relevant characteristics of participants, documents, or events included in the study; level of participation (could be reported in results)	NA in protocol
**Data processing** - Methods for processing data prior to and during analysis, including transcription, data entry, data management and security, verification of data integrity, data coding, and anonymization/de-identification of excerpts	11
**Data analysis** - Process by which inferences, themes, etc., were identified and developed, including the researchers involved in data analysis; usually references a specific paradigm or approach; rationale**	11
**Techniques to enhance trustworthiness** - Techniques to enhance trustworthiness and credibility of data analysis (e.g., member checking, audit trail, triangulation); rationale**	8

Results/findings

**Synthesis and interpretation** - Main findings (e.g., interpretations, inferences, and themes); might include development of a theory or model, or integration with prior research or theory	NA in protocol
**Links to empirical data** - Evidence (e.g., quotes, field notes, text excerpts, photographs) to substantiate analytic findings	NA in protocol

Discussion

**Integration with prior work, implications, transferability, and contribution(s) to the field** - Short summary of main findings; explanation of how findings and conclusions connect to, support, elaborate on, or challenge conclusions of earlier scholarship; discussion of scope of application/generalizability; identification of unique contribution(s) to scholarship in a discipline or field	Discussion of potential study significance 13-15
**Limitations** - Trustworthiness and limitations of findings	14

Other

**Conflicts of interest** - Potential sources of influence or perceived influence on study conduct and conclusions; how these were managed	15
**Funding** - Sources of funding and other support; role of funders in data collection, interpretation, and reporting	15

*The authors created the SRQR by searching the literature to identify guidelines, reporting standards, and critical appraisal criteria for qualitative research; reviewing the reference lists of retrieved sources; and contacting experts to gain feedback. The SRQR aims to improve the transparency of all aspects of qualitative research by providing clear standards for reporting qualitative research.

For peer review only - http://bmjopen.bmj.com/site/about/guidelines.xhtml

**The rationale should briefly discuss the justification for choosing that theory, approach, method, or technique rather than other options available, the assumptions and limitations implicit in those choices, and how those choices influence study conclusions and transferability. As appropriate, the rationale for several items might be discussed together.

Reference:

O'Brien BC, Harris IB, Beckman TJ, Reed DA, Cook DA. **Standards for reporting qualitative research: a synthesis of recommendations.** *Academic Medicine*, Vol. 89, No. 9 / Sept 2014 DOI: 10.1097/ACM.0000000000000388

For peer review only

StaRI
Standards for reporting implementation studies

Standards for Reporting Implementation Studies: the StaRI checklist for completion

The StaRI standard should be referenced as: Pinnock H, Barwick M, Carpenter C, Eldridge S, Grandes G, Griffiths CJ, Rycroft-Malone J, Meissner P, Murray E, Patel A, Sheikh A, Taylor SJC for the StaRI Group. Standards for Reporting Implementation Studies (StaRI) statement. *BMJ* 2017;356:i6795

The detailed Explanation and Elaboration document, which provides the rationale and exemplar text for all these items is: Pinnock H, Barwick M, Carpenter C, Eldridge S, Grandes G, Griffiths C, Rycroft-Malone J, Meissner P, Murray E, Patel A, Sheikh A, Taylor S, for the StaRI group. Standards for Reporting Implementation Studies (StaRI). Explanation and Elaboration document. *BMJ Open* 2017 2017;7:e013318

Notes: A key concept of the StaRI standards is the dual strands of describing, on the one hand, the implementation strategy and, on the other, the clinical, healthcare, or public health intervention that is being implemented. These strands are represented as two columns in the checklist.

The primary focus of implementation science is the implementation strategy (column 1) and the expectation is that this will always be completed.	The evidence about the impact of the intervention on the targeted population should always be considered (column 2) and either health outcomes reported or robust evidence cited to support a known beneficial effect of the intervention on the health of individuals or populations.

The StaRI standardsrefers to the broad range of study designs employed in implementation science. Authors should refer to other reporting standards for advice on reporting specific methodological features. Conversely, whilst all items are worthy of consideration, not all items will be applicable to, or feasible within every study.

Checklist item		Reported on page #	Implementation Strategy	Reported on page #	Intervention
			"Implementation strategy" refers to how the intervention was implemented		"Intervention" refers to the healthcare or public health intervention that is being implemented.
**Title and abstract**					
Title	**1**	1	Identification as an implementation study, and description of the methodology in the title and/or keywords		
Abstract	**2**	2-4	Identification as an implementation study, including a description of the implementation strategy to be tested, the evidence-based intervention being implemented, and defining the key implementation and health outcomes.		
**Introduction**					
Introduction	**3**	2-3<br>12-13	Description of the problem, challenge or deficiency in healthcare or public health that the intervention being implemented aims to address.		
Rationale	**4**	6-7	The scientific background and rationale for the implementation strategy (including any underpinning theory/framework/model, how it is expected to achieve its effects and any pilot work).	3	The scientific background and rationale for the intervention being implemented (including evidence about its effectiveness and how it is expected to achieve its effects).

For peer review only

Aims and objectives	**5**	4	The aims of the study, differentiating between implementation objectives and any intervention objectives.		
**Methods: description**					
Design	**6**	4-11	The design and key features of the evaluation, (cross referencing to any appropriate methodology reporting standards) and any changes to study protocol, with reasons		
Context	**7**	*7 (see text below)	The context in which the intervention was implemented. (Consider social, economic, policy, healthcare, organisational barriers and facilitators that might influence implementation elsewhere).		
Targeted 'sites'	**8**	4	The characteristics of the targeted 'site(s)' (e.g locations/personnel/resources etc.) for implementation and any eligibility criteria.	4	The population targeted by the intervention and any eligibility criteria.
Description	**9**	*9 (see text below	A description of the implementation strategy	*9 (see text below	A description of the intervention
Sub-groups	**10**	NA	Any sub-groups recruited for additional research tasks, and/or nested studies are described		
**Methods: evaluation**					
Outcomes	**11**	*11 (see text below	Defined pre-specified primary and other outcome(s) of the implementation strategy, and how they were assessed. Document any pre-determined targets	*11 (see text below	Defined pre-specified primary and other outcome(s) of the intervention (if assessed), and how they were assessed. Document any pre-determined targets
Process evaluation	**12**	*12 (see text below	Process evaluation objectives and outcomes related to the mechanism by which the strategy is expected to work		
Economic evaluation	**13**	11	Methods for resource use, costs, economic outcomes and analysis for the implementation strategy	11	Methods for resource use, costs, economic outcomes and analysis for the intervention
Sample size	**14**	*14	Rationale for sample sizes (including sample size calculations, budgetary constraints, practical considerations, data saturation, as appropriate)		
Analysis	**15**	10-11	Methods of analysis (with reasons for that choice)		

Sub-group analyses	**16**	NA	Any a priori sub-group analyses (e.g. between different sites in a multicentre study, different clinical or demographic populations), and sub-groups recruited to specific nested research tasks

*7 – data to provide a description of the context will be gathered through Research Activity (RA) RA1 – staff interviews
*9– full description of the intervention and implementation strategy will be described in the final write up of the research and will be informed by RA 1 & 7
*11– the outcome of the implementation strategy and intervention will be described in the final write up of the research and will be informed by RA 2-7 and service evaluation/NHS friends and family test data
*12 – process evaluation will be informed by RA1 (logic model development), RA2-7 (process measures)
*14 – sample size will be informed by the study as an aspect of feasibility

**Results**					
Characteristics	**17**	*17	Proportion recruited and characteristics of the recipient population for the implementation strategy	*17	Proportion recruited and characteristics (if appropriate) of the recipient population for the intervention
Outcomes	**18**	As *11	Primary and other outcome(s) of the implementation strategy	As *11	Primary and other outcome(s) of the Intervention (if assessed)
Process outcomes	**19**	As *12	Process data related to the implementation strategy mapped to the mechanism by which the strategy is expected to work		
Economic evaluation	**20**	As described pg 11	Resource use, costs, economic outcomes and analysis for the implementation strategy	As described pg 11	Resource use, costs, economic outcomes and analysis for the intervention
Sub-group analyses	**21**	NA	Representativeness and outcomes of subgroups including those recruited to specific research tasks		
Fidelity/ adaptation	**22**	*22	Fidelity to implementation strategy as planned and adaptation to suit context and preferences	*22	Fidelity to delivering the core components of intervention (where measured)

For peer review only - http://bmjopen.bmj.com/site/about/guidelines.xhtml

Contextual changes	**23**	*23	Contextual changes (if any) which may have affected outcomes		
Harms	**24**	*24	All important harms or unintended effects in each group		
**Discussion**					
Structured discussion	**25**	NA at protocol stage	Summary of findings, strengths and limitations, comparisons with other studies, conclusions and implications		
Implications	**26**	NA at protocol stage	Discussion of policy, practice and/or research implications of the implementation strategy (specifically including scalability)	NA at protocol stage	Discussion of policy, practice and/or research implications of the intervention (specifically including sustainability)
**General**					
Statements	**27**	14-15	Include statement(s) on regulatory approvals (including, as appropriate, ethical approval, confidential use of routine data, governance approval), trial/study registration (availability of protocol), funding and conflicts of interest		

*17 – participant demographics will be collected for all RA (including participation in the intervention via RA 4 and reported
*22 – Fidelity and adaptation will be described through comparison of RA1, RA1 cont. and RA7, and through RA2
*23 Contextual changes will be described through data collection in RA1 and RA7, and RA2
*24 Harms/unintended effects data will be collected in RA3, RA4, RA6, RA7

For peer review only
